# Substantial heterogeneity in trauma triage tool characteristic operationalization for identification of major trauma: a hybrid systematic review

**DOI:** 10.1007/s00068-024-02694-6

**Published:** 2025-01-24

**Authors:** N. A. Donnelly, L. Brent, P. Hickey, S. Masterson, C. Deasy, J. Moloney, M. Linvill, R. Zaidan, A. Simpson, Frank Doyle

**Affiliations:** 1 RCSI University of Medicine and Health Sciences, Dublin, Ireland; 2 National Office of Clinical Audit, Dublin, Ireland; 3 National Ambulance Service, Health Service Executive, Dublin, Ireland; 4 Cork University Hospital, Cork, Ireland; 5 University College Cork, Cork, Ireland

**Keywords:** Trauma, Triage, Systematic review, Clinical prediction rule

## Abstract

**Purpose:**

Trauma Triage Tools (TTTs) support pre-hospital staff to identify major trauma patients based on prehospital characteristics and bring them to appropriate trauma centres. However, while triaging trauma has been examined extensively, there appears to be little consensus on how variables within TTTs are applied. We therefore aimed to examine the prehospital characteristics and their operationalization applied in the international literature in TTTs.

**Methods:**

We applied a hybrid systematic review approach. Searches were conducted in multiple databases. We initially searched for systematic reviews that analyse prehospital characteristics applied in TTTs, then supplemented this with an updated search of original TTT papers from November 2019.

**Results:**

We identified 92 papers which identified 52 adult general population TTTs. Results indicate considerable heterogeneity in prehospital characteristics included in TTTs internationally. There was similarity in the higher-level categories included in the tools: tools often included measurements of a patient’s physiological characteristics, injury characteristics, mechanism of injury and any modifiers for high-risk groups. However, the prehospital characteristics that made up those groups, how they were applied and interpreted were found to vary considerably.

**Conclusion:**

While there is agreement in the higher-level categories used in TTTs, the thresholds adopted in specific variables vary widely, which may reflect statistical rather than clinical considerations. This may contribute to considerable variation in standards of major trauma triaging internationally. An agreed taxonomy of operationalization of prehospital characteristics used in TTTs is required to prevent sub-optimal clinical decision-making in major trauma triaging.

**Registration:**

PROSPERO CRD42023393094.

**Supplementary Information:**

The online version contains supplementary material available at 10.1007/s00068-024-02694-6.

## Introduction

Trauma is a leading cause of morbidity and mortality internationally [1, 2]. It is estimated that, on an annual basis, 6 million people die as consequence of traumatic injuries, accounting for 10% of deaths globally [3]. For those aged under 35 trauma is the leading cause of death and disability [4]. There is a considerable and growing body of international evidence which demonstrates significant improvements both in the trauma care process and outcomes for patients through re-configuring trauma care services from that which is fragmented to fully integrated inclusive trauma networks [5]. Such networks provide for the continuum of trauma complexity, with a patient brought to a range of differing services (Major Trauma Centres, Trauma Units with specialist services, Local Emergency Hospitals and Local Injury Units) depending on the severity of their injuries. Studies from the UK, USA and Australia suggest trauma networks reduce mortality by 15–25%, shortens hospital length of stay by an average of four days, lower the odds of readmission to hospital and are cost-effective [6–10].

A backbone of any trauma network is a trauma triage tool (TTT). TTTs are designed to support clinical decision making in the prehospital setting. In the context of major trauma, this involves intense, time-sensitive, and complex care. TTTs support prehospital staff (emergency medical technicians, paramedics, advanced paramedics and doctors) to rule out low-risk patients and identify high-risk major trauma patients that would benefit from care at a major trauma center. Such support is of substantial added-value in the trauma care process. Thus, a trauma triage tool is often regarded as an integral part of an integrated trauma-care pathway, from incident to hospital discharge [11]. The accuracy of a TTT is based on the tool’s ability to correctly identify those with and without severe injuries. Under-triage occurs when patients with severe injuries are incorrectly brought to a lower-level facility. This is associated with increased mortality, delayed diagnosis, and decreased functional outcomes [12]. Conversely, over-triage occurs when patients with less severe injuries are admitted to higher level care facilities, resulting in overburdening major trauma centres and unnecessary resource use [13]. The American College of Surgeons Committee on Trauma (ACS-COT) recommends acceptable over-triage rates of 25–35% and under-triage of 5% [14]. There is considerable international literature on the development of triage tools that have aimed at reducing over and under-triage by standardizing triage criteria in order to identify major trauma patients that would benefit from care in a major trauma center. However, a number of systematic reviews in the area have found the current triage tools do not meet international guidance for acceptable over or under-triage rates [12, 15–17]. Consequently, there is no consensus on an optimal triage tool [12] and with that, no consensus on the minimum criteria for prehospital identification of major trauma [17].

In tandem with this, while there is considerable literature on the development of triage tools aiming to improve the identification of major trauma patients, recent research has suggested that where there are multiple similar models in the development of such tools, there may be a combination of selective reporting, selective variable inclusion, modifying variable thresholds, re-analysis of the data and/or ‘cherry picking’ statistically significant results to develop tools based on models with more favorable abilities to discriminate higher-risk patients [18, 19]. Examining and synthesizing the prehospital characteristics applied to identify major trauma patients could better inform the development of future trauma triage tools by identifying best practice for same. We therefore aimed to document the range of variables included in TTTs and explore the heterogeneity in the operationalization of the adopted prehospital characteristics included in the TTTs.

## Objective

Examine the prehospital characteristics and their operationalization applied in the international literature in TTTs.

## Methods

### Study design

This review has been pre-registered (PROSPERO: CRD42023393094) and a detailed full protocol published (40). As several systematic reviews have examined the degree to which different triage tools successfully identify major trauma patients, we conducted a hybrid review, which involves a combination of overview of reviews and individual systematic review techniques [20–23]. We have reported the conduct of this review in accordance with the Preferred Reporting Items for Systematic Review and Meta-Analysis (PRISMA) updated guidelines [24]. The completed PRISMA Checklist can be found in Appendix 1.

For this hybrid approach, two search strategies were employed. Firstly, we searched for all systematic reviews on TTTs, not limited by year of publication (from inception to 31st January 2023), and identified individual studies on TTTs cited within these reviews. We extracted the relevant data both in the systematic reviews and the original papers on the prehospital characteristics that were included in individual TTTs. This was then supplemented with a second search for original papers on TTTs, published from November 2019, the date of the search in the most recently published systematic review in the area [11].

### Eligibility criteria for selecting studies

#### Studies

Studies that were eligible to be included in the review were papers on TTTs that aimed to identify major trauma patients. To identify these papers, we first searched for systematic reviews on TTTs. We used these systematic reviews to identify the studies to be included in the review. These systematic reviews were not limited by year of publication or destination to which trauma patients were brought. We also identified recent papers (published from November 2019, the date of the search in the most recent review in the area) that analyse prehospital characteristics associated with major trauma patients. Only English language papers were included. We excluded studies of triage outside of the prehospital setting, studies concerned with mass casualty trauma events, in-hospital trauma team response, and studies only concerned with activation of helicopter response.

#### Participants

We included studies on TTTs conducted for the general adult population. Patients with medical needs that are not the direct result of an injury were excluded. Studies which were confined to a specific cohort of trauma patients, namely pediatric and older patient populations, were excluded from the data synthesis. There were a number of reasons for this. Firstly, as the review was conducted to inform the development of a clinical prediction tool for the general trauma patient population, it was important that the evidence informing the development of the clinical prediction tool was appropriate to the patient population to which it would be applied. In tandem with this, pediatric and older patient populations have differing physiological characteristics, for example the general systolic blood pressure and respiratory rate for young children and adults over the age of 65 are considerably different [25–29]. These groups also often present with specific and complex care needs. Thus, there is a considerable and growing body of literature to suggest trauma triaging in pediatric or older adult populations should include criteria that is more sensitive to the needs of those populations [25–29].

#### Variables of interest

Prehospital characteristics applied in trauma triage tools that are associated with major trauma, and their specific operationalizations (e.g. thresholds adopted), were the outcome of interest. These characteristics included, but were not limited to, patient characteristics (age, sex etc.), physiologic characteristics (blood pressure, respiratory rate, Glasgow Coma Score [30] etc.), mechanism of injury (fall, motor vehicle accident etc.), injury characteristics (penetrating or blunt force injury, body region(s) injured etc.). Medical needs that are not the direct result of an injury (e.g., diabetes) were excluded. Triage tools that did not apply a standardized approach to assess prehospital characteristics, or if the characteristics were not described in the tool, were excluded.

### Information sources

#### Databases

Both of the searches for systematic reviews and original papers were conducted in the following databases: Ovid MEDLINE, Embase, Cochrane Library of Systematic Reviews and Cochrane Central Register of Clinical Trials.

#### Search strategy

The search strategy was created with the support of an Information Specialist in RCSI University of Medicine and Health Sciences (AS) who is experienced in database searching and systematic review search strategy refinement. The database search included a search of subject headings, keywords and associated synonyms. The search terms were: trauma, trauma centers, trauma system; triage, undertriage, overtriage; systematic review, meta-analysis (only when searching specifically for systematic reviews). The exact search strategy for each database can be found in Appendix 2.

### Data collection and synthesis of results

#### Selection process

Results were imported to EndNote software and duplicate articles were removed. Two reviewers (ML and RZ) undertook duplicate screening of titles and abstracts of papers identified by the literature search. Papers that did not meet the inclusion criteria were excluded. Disagreements were discussed with a third reviewer (ND). All papers identified as potentially relevant were retrieved and read in full to determine eligibility for inclusion.

#### Data extraction

A pre-defined data extraction template was applied. Data was extracted to Microsoft Excel. Extracted data included the study author(s), year of publication, country, name of the triage tool or protocol, study population, sample size, study design, measures of major trauma applied in the study and prehospital characteristics used to identify major trauma patients.

##### Synthesis of results

The objective of the review was to examine the prehospital characteristics and their operationalization in the international literature in TTTs. In particular, to comprehensively identify the prehospital characteristics and the thresholds applied in those tools that identify major trauma patients. Therefore, narrative synthesis was the most appropriate method to analyse the results. We categorized results and presented findings using a traffic light system for ease of interpretation. That is, where 60% or more of the tools included a variable, this is presented in green (bold font). Where 30–59% of the tools included the variable, this is presented in orange (normal font). If less than 30% of the tools included the variable, this is presented in in red (*italics*).

## Results

### Study selection

**Fig. 1:**
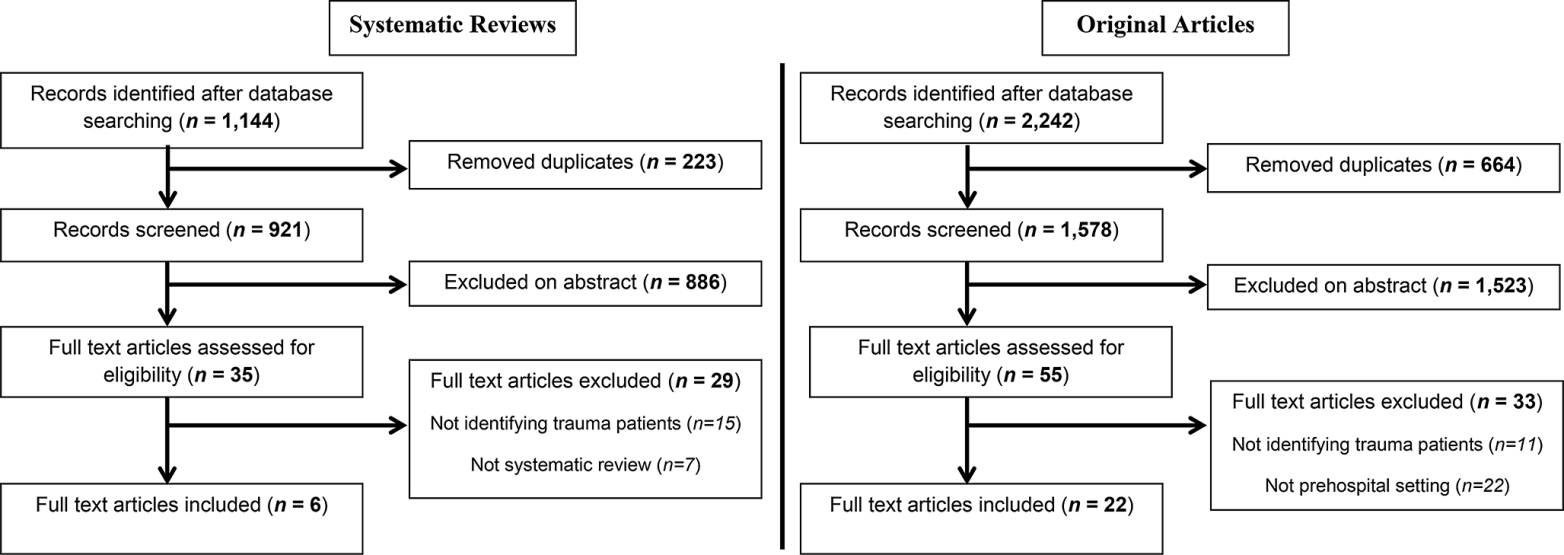
Flow diagrams of records identified through the separate database searching

As two separate search strategies were employed, Fig. 1 below presents two flow diagrams, one for each search strategy. The study selection process for each will be described in turn. Starting with the search for systematic reviews. The database search identified 1,144 records. There were 223 duplicates which were removed. Following abstract screening, 886 articles were excluded. Thirty-five full text articles were assessed for eligibility, from which 29 were excluded (15 did not identify trauma patients, 7 were not systematic reviews, 2 were specific to pediatric populations and 5 were specific to older patient populations). Thus, 70 individual papers from 6 systematic reviews were included in the analysis.

The database search for original articles, post November 2019, identified 2,242 records. From these records, 664 duplicates were removed. Abstract screening identified 1,523 records which did not meet the inclusion criteria and so were removed. A further 55 full text articles were assessed for eligibility, from which 33 articles were excluded (11 did not identify trauma patients and 22 were not in a prehospital setting). This left 22 full text articles to be included in the analysis. Therefore, between the systematic reviews which identified 70 individual papers and the 22 original articles, data from a total of 92 studies were included in the analysis.

### Study characteristics

The characteristics of 92 included studies are presented in Appendix 3. Studies were conducted in Australia, Canada, Denmark, France, China, Italy, Norway, Spain, Sweden, Thailand, The Netherlands, United Kingdom, United States, and Uganda. 49% of all studies were from the United States. Publication dates ranged from 1986 to 2023. An average of 2–3 studies a year were published between 1986 and 2009, publications numbers gradually increased overtime (4 in 2010, 9 in 2011, 12 in 2014 and 14 in 2016). In the majority of cases, the study design applied was retrospective (77% of studies). Sample sizes ranged considerably from 130–4,152,541. The average sample size was 130,514 participants.

The majority of studies (*n* = 56) applied the Injury Severity Score (ISS) to define major trauma patients [31]. In 14 of these cases, the ISS was used in combination with other measures. These measures included in-hospital mortality, intensive care admission and urgent surgery. After the ISS, the second most common outcome measure applied was patient mortality, which was measured in 21 studies. It should be noted that the measurement of mortality varied from death in the Emergency Department/Emergency Room, death within 24 h of presentation to hospital, death within 8 days, death within 30 days or mortality from any cause during hospital stay. Other outcome measures identified in the studies included Mortality and Trauma and Injury Severity Score (TRISS) [32] and the New Injury Severity Score (NISS) [33].

The 92 studies identified 52 different adult/general population trauma triage tools. The most commonly applied tool was the Field Triage Decision Scheme by The American College of Surgeons Committee on Trauma (ACS-COT), which was applied in 35 publications (see Appendix 3). The American College of Surgeons Committee on Trauma update the triage decision scheme every couple of years, taking into account updated evidence. Thus, given the time span of included publications in the review (1986–2023), a number of versions of the tool were identified [14, 34–37]. In the articles identified, the tool was largely applied in the United States, but it was also applied to analysis in Denmark, the Netherlands and Italy. The second most commonly applied tool was the Triage Revised Trauma Score (T-RTS) [38] which was applied in 8 studies from a number of countries (France, the United Kingdom, United States, the Netherlands and Spain). The original Trauma Score was applied to the analysis of 6 of the identified studies [38, 39]. All 6 of these studies were conducted in the United States.

### Synthesis of results

Tables 1, 2, 3 and 4 below present the synthesized results of the prehospital characteristics included in the 52 identified trauma triage tools. As described above, this has been presented using a traffic light system ( > = 60% inclusion as green, 30–59% as orange, < 30% as red). The tables present the proportion of tools with the variable groups and the sub-categories within each group. The raw data which make up these tables is provided in Appendix 4 (Raw Data: Master variables by category sheet).

**Table 1:**
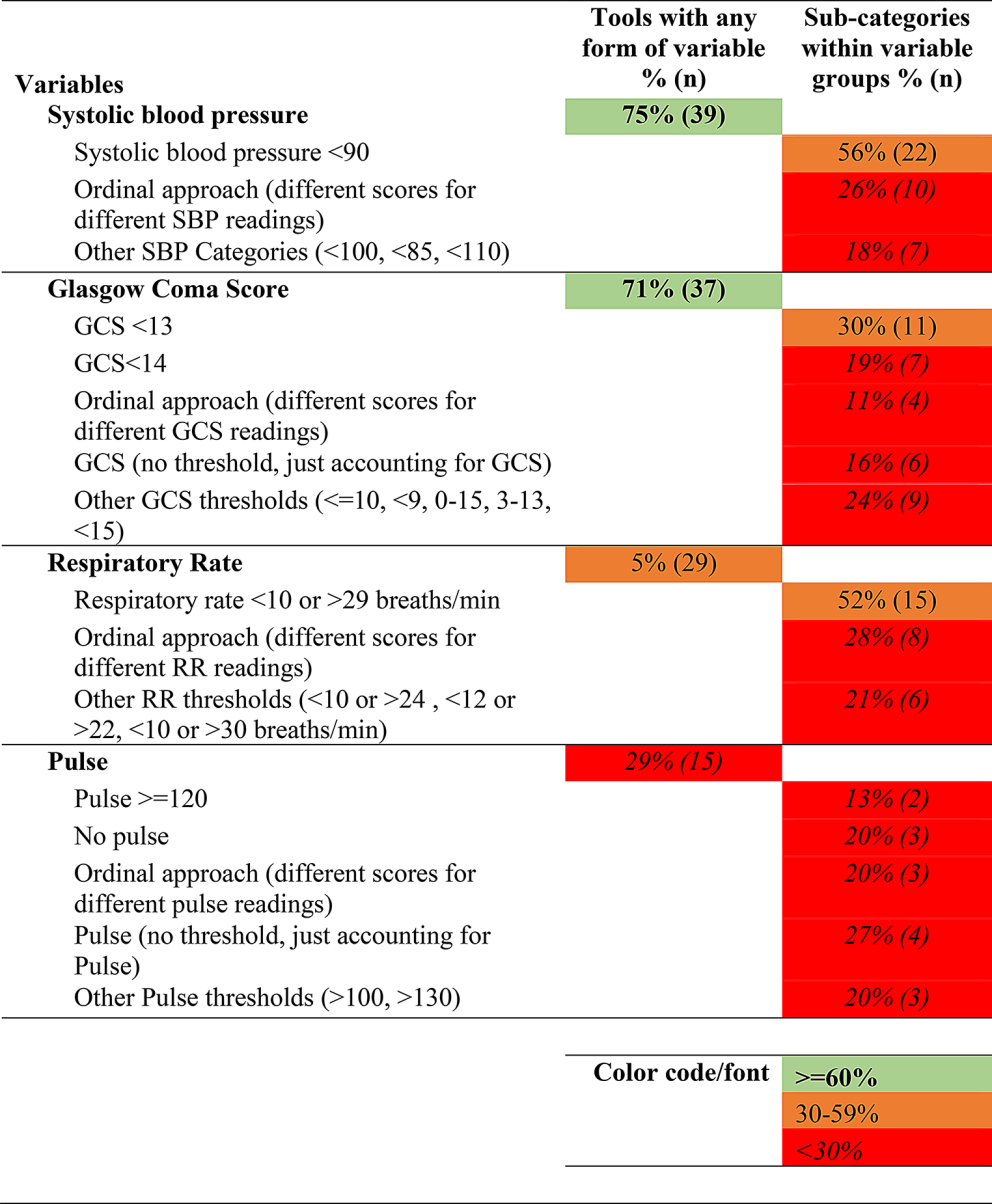
Physiologic characteristics identified in trauma triage tools

**Table 2:**
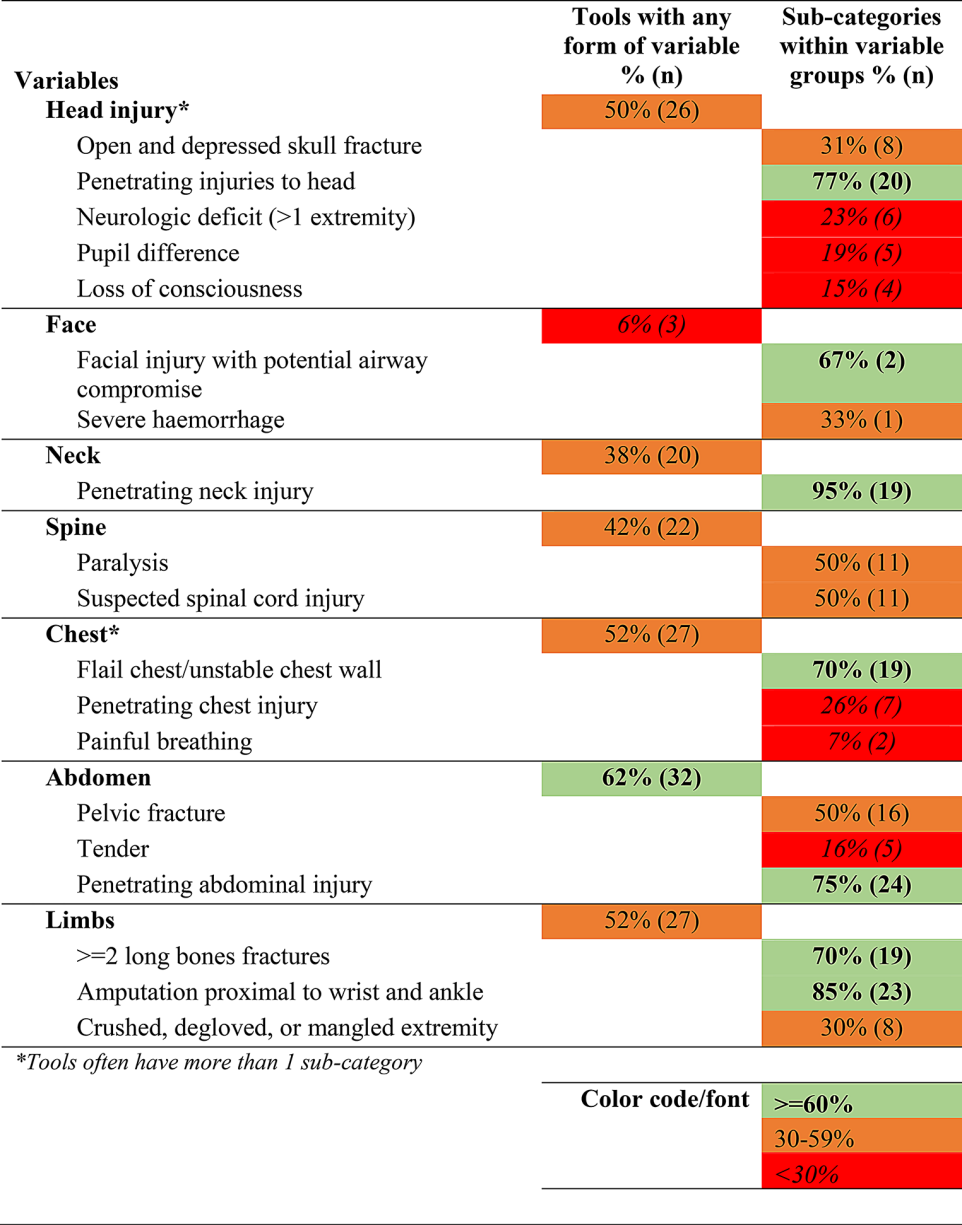
Injury characteristics identified in trauma triage tools

**Table 3:**
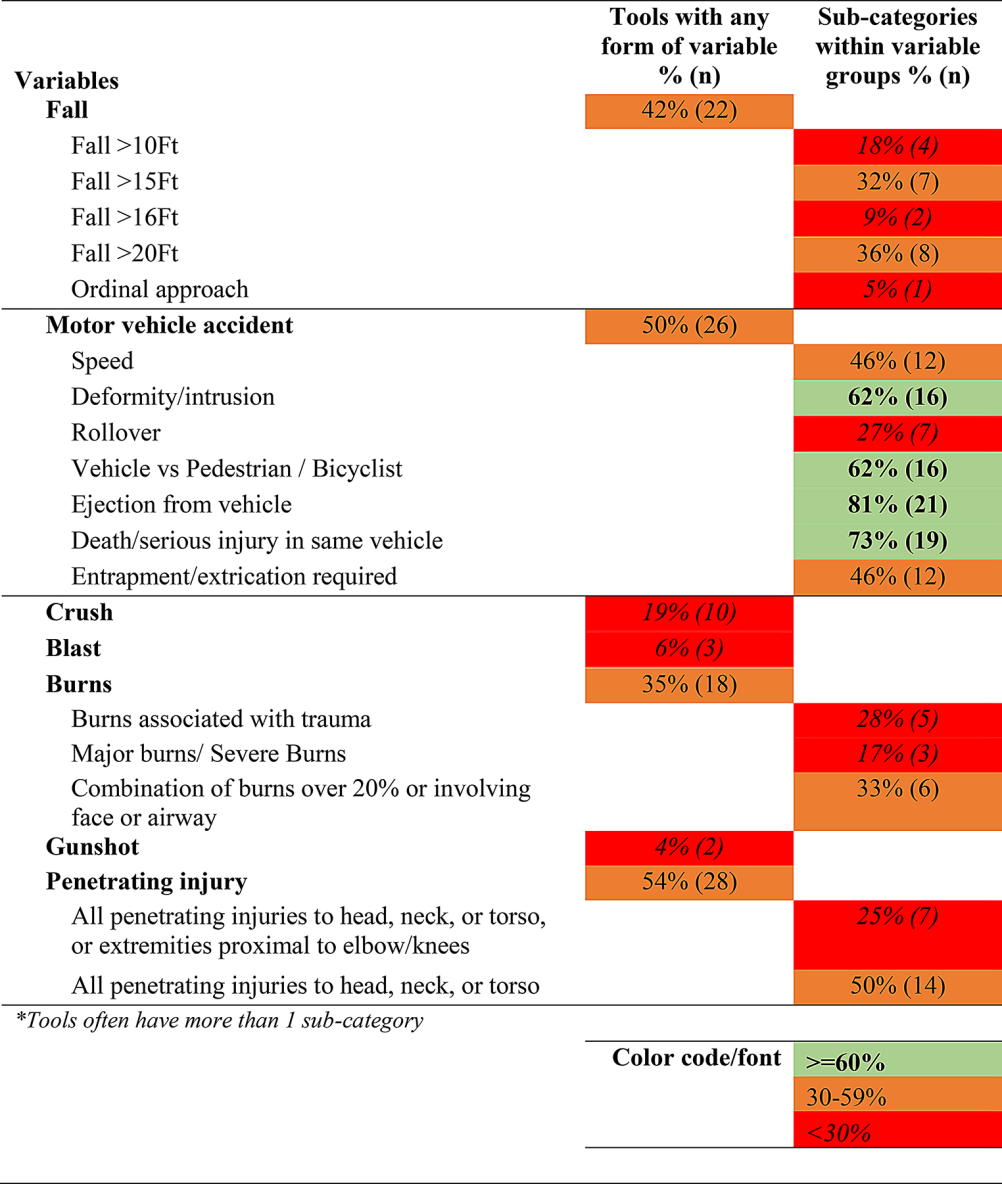
Mechanism of injury characteristics identified in trauma triage tools

**Table 4:**
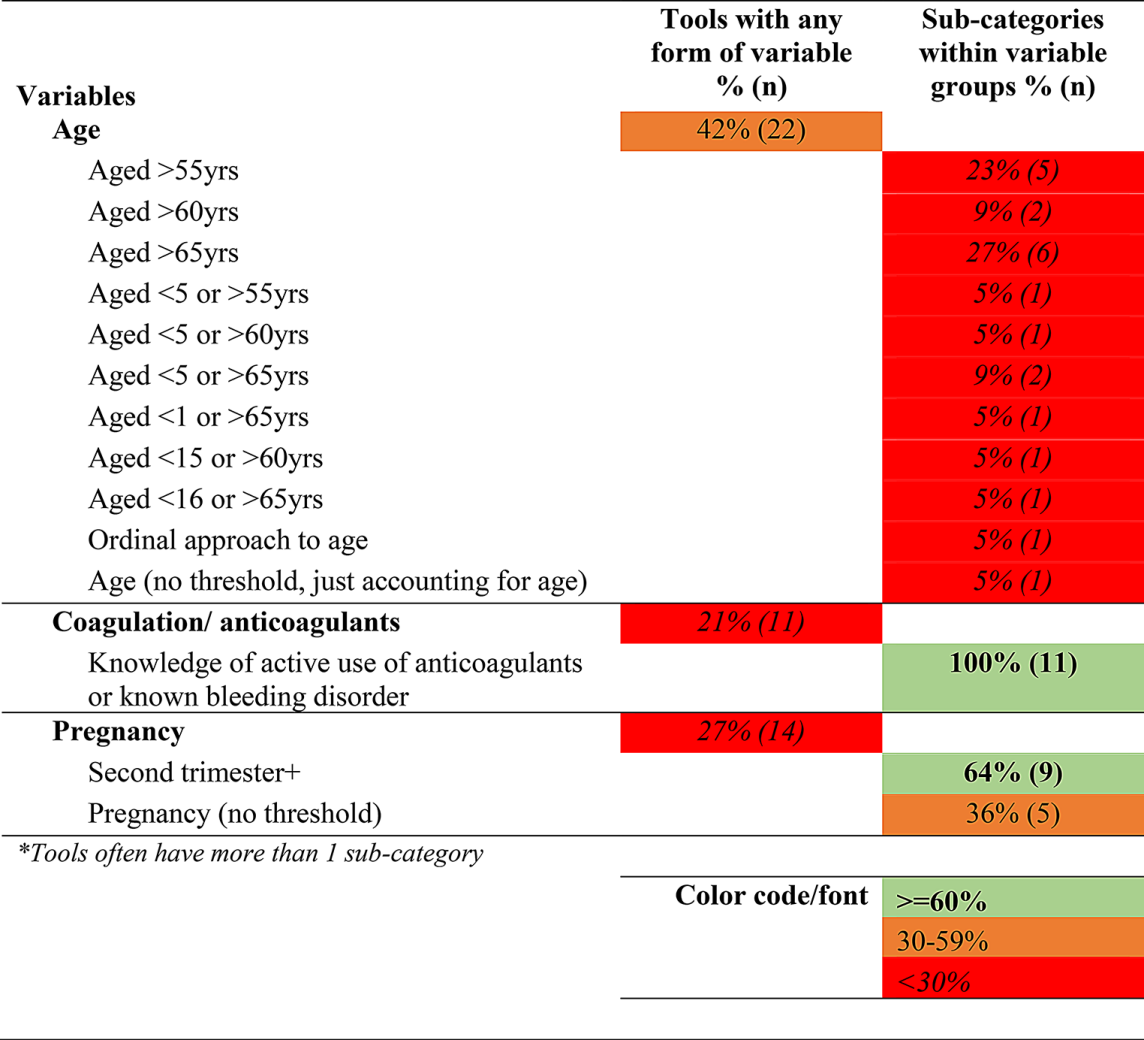
Modifiers for high-risk groups identified in trauma triage tools

There appears to be a broad overlap in the higher-level categories of prehospital characteristics included in TTTs. It would appear that most TTTs account for a patient’s physiological characteristics, injury characteristics, the mechanism of injury and then modifiers for high-risk groups. However, there is considerable variation between TTTs in the make-up and operationalization of these groups and individual variables.

Physiological characteristics were the most commonly included category, with 90% of triage tools accounting for a patient’s physiological characteristics (presented in Table 1 below). While 75% of tools account for a patient’s systolic blood pressure, we found a substantial range in how this is categorized and interpreted. Indeed, 25 different ways of categorizing and threshold interpretations for systolic blood pressure were identified amongst the tools. Of the tools that accounted for systolic blood pressure, the most commonly applied threshold was systolic blood pressure < 90 mm HG, applied in 56% of tools (*n* = 22). One in four tools applied an ordinal approach to systolic blood pressure, with differing scores applied to different systolic blood pressure readings, each of which varied across the 10 different tools that applied such a scoring system. We found 71% of tools account for a patient’s Glasgow Coma Score (GCS). However, as with systolic blood pressure, we found GCS was categorized and interpreted in range of ways. For example, in 30% of tools the GCS threshold was < 13, while in 19% the threshold was < 14. A range of different thresholds were applied in a further 24% of tools (see Table 1).

Following physiological characteristics, the second most commonly applied category was injury characteristics, applied in 69% of tools. The results for which are presented in Table 2. We found less agreement in how injury characteristics are accounted for than was found for physiological characteristics. This was observed both in terms of the proportion of tools that account for different injury groups and the sub-categories of injuries included. For example, 50% of the identified TTTs included head injuries. However, the means of categorizing head injuries varied. Amongst the 52 trauma triage tools identified, we found 33 different ways that head injuries were categorized. Of those tools that accounted for head injuries, 77% included penetrating injuries to the head. Open and depressed skull fractures were observed in 31%. The other frequent means of categorizing head injuries included neurologic deficit in more than 1 extremity (included in 23%), pupil difference (19%) and loss of consciousness (15%).

We found that 62% of TTTs (*n* = 32) considered whether a patient had an abdominal injury. For most of these tools, this centered on penetrating injuries to the abdomen (75% *n* = 24) or pelvic fractures (50%, *n* = 16). Injuries to a patient’s limbs were accounted for in 52% (*n* = 27) of TTTs. In the majority of tools this concerned two or more long bone fractures (*n* = 19 tools) or amputation proximal to wrist or ankle (*n* = 23 tools).

The mechanism of injury was accounted for in 56% of TTTs, presented in Table 3 below. These mechanisms included injuries as a result of a fall, motor vehicle accident, crush, blast, gunshot or burns. 42% of tools took falls into account. However, these tools applied a range of different thresholds for falls, such as falls over 10 feet (*n* = 4), over 15 feet (*n* = 7), over 16 feet (*n* = 2), or over 20 feet (*n* = 8).

Injuries as a result of a motor vehicle accident were accounted for in 50% of identified tools. We found the measurement of elements of a motor vehicle accident presented the greatest degree of variation amongst the tools, with 81 different ways of measuring aspects of a motor vehicle accident. Often there was slight variation in the thresholds applied, for example extrication time of more than 20 min, or more than 30 min, or prolonged extrication, or entrapped in the vehicle, entrapped elsewhere, entrapped with intrusion, entrapped with compression. The measurements of speed also varied from more than 32KM/h, more than 60KM/h, more than 65KM/h, more than 70KM/h, ‘high-speed’, ‘global assessment of speed’. We found a variety of ways of accounting for motor vehicle and bicyclist accidents, such as: ‘auto vs bicyclist’, ‘auto vs bicyclist with significant impact’, ‘auto vs bicyclist thrown, run over or > 20mph impact’, ‘motor vehicle vs bicycle impact > 10km/h’. Similarly, while 62% (*n* = 16) of tools that accounted for motor vehicle accidents included some measurements of intrusion or deformation of the vehicle, there was also variation in the ways that this was measured. For example, ‘vehicle intrusion’ ‘intrusion occupant side > 12 inches, 18 inches in any site in car’, ‘intrusion into occupant compartment > 30 cm’, ‘passenger space intrusion > 12 inches’, ‘structural intrusion into passenger compartment > 20 inches’, ‘vehicle deformation’, ‘vehicle deformity > 50 cm’. There were elements to a motor vehicle accident that were found to be more consistently and frequently included in the tools. In particular, ejection from a vehicle (measured in 21 tools) and death or serious injury in a vehicle (measured in 19 tools).

The final broad category found in the tools identified was modifiers for high-risk groups, presented in Table 4 below. These modifiers included a patient’s age, knowledge of anticoagulant use or known bleeding disorders and pregnancy. Amongst the 52 identified tools, 22 (42%) took a patient’s age into account. However, we found considerable variation in how age was categorized, with 11 different approaches identified. This included differing thresholds for older patients (over 55 years of age, 60 years or 65 years of age), or thresholds for both younger and older patients (less than 5 to over 55 years of age, less than 1 to over 65 years of age etc.). Interestingly, only two of the tools that accounted for age were published in the 1980s, the rest were published after 2001.

## Discussion

### Summary of evidence

This is the first review to profile the most commonly used variables in TTTs and highlight the heterogeneity of variable operationalization within these. We identified 92 different articles and 52 trauma triage tools. Such a wide range of articles and TTTs meant we could comprehensively establish the range of prehospital characteristics that have been applied to identify major trauma patients. The study results suggest that there is considerable heterogeneity in prehospital characteristics included in trauma triage tools internationally. We found similarity in the higher-level categories included in the tools. That is, tools often included measurements of a patient’s physiological characteristics, injury characteristics, mechanism of injury and any modifiers for high-risk groups. However, the prehospital characteristics that make up those categories, how they are categorized and how they are interpreted were found to vary considerably between the tools identified. Given the substantial differences found between the tools, especially thresholds included, the results indicate considerable variation in standards of major trauma triaging internationally.

### Variation in the prehospital characteristics applied to identify major trauma patients

We found extensive diversity in the prehospital characteristics applied to identify major trauma patients. We observed numerous ways to categorize essentially the same prehospital characteristic with multiple yet slightly differing thresholds for the variables. For example, while 75% of the identified tools accounted for a patient’s systolic blood pressure, we found the tools presented 25 different ways of categorizing and threshold interpretations for systolic blood pressure. Similarly, we found considerable variation in the thresholds applied in the context of motor vehicle accidents. This included variation in thresholds for extrication time, speed, vehicle intrusion and motor vehicle vs. bicycle impact.

The extensive variation in the thresholds applied in the identified tools is of concern. It would appear to mirror the suggestion from recent research that where there are multiple similar models in the development of such clinical prediction tools, there may be a combination of selective reporting, re-categorization and re-analysis of the data and/or handpicking statistically significant results to develop tools that are based on models with more favorable abilities to identify high-risk patients [18, 19]. For example, White and colleagues analyzed the distribution of the areas under the receiver operating curve (AUC) for clinical prediction tools [19]. The AUC is a measure used to describe the ability of a clinical prediction tool to discriminate between patients with greater risks of an outcome of interest. The ability of the clinical prediction tool to distinguish higher risk patients is conventionally interpreted through AUC threshold values of 0.7, 0.8 and 0.9 as being acceptable, fair-good and excellent. However, such thresholds may encourage goals which researchers try to achieve [19]. White and colleagues found the distribution of AUC values from PubMed abstracts were clearly in excess of the conventional 0.7, 0.8 and 0.9 thresholds, suggesting an over-inflation of AUC values [19]. Re-analyzing data or selective reporting to publish clinical prediction tools with more favorable abilities to identify high-risk patients’ would risk exposing patients to sub-optimal clinical decision-making [19]. It would be of concern should this be happening in the trauma triage literature.

An alternative explanation could relate to the fact that various population and environmental characteristics can have significant effects of the variables that are ultimately included in TTTs and whether these are then externally valid to other areas/populations. Examples include automotive safety regulations (e.g. speed limits), popularity or otherwise of different transport modes (e.g. cycling), base rates of major trauma, age and so on (e.g [12, 40, 41]). This of course means that there can often be justifiable heterogeneity in variable categorization within TTTs– however it is debatable whether the extent of heterogeneity in our results is explained by these other important factors.

### Strengths and limitations

The hybrid approach to the review enabled an extensive literature search with the identification of a considerable number of articles. This gave the study significant strength.

The study focused on the prehospital characteristics applied in trauma triage tools. Studies that analysed prehospital characteristics of major trauma patients but did not develop a TTT were excluded. Potentially this may have resulted in the omission of some prehospital characteristics. However, given the decades of research in the articles included in the analysis, spanning 1986–2023, it is unlikely that pertinent variables have been omitted from TTTs included in the review. Therefore, we do not anticipate a significant impact on the study findings given the omission of such studies.

We excluded tools that are confined to pediatric and older patient populations as these groups often present with complex care needs and given the concerns raised in the growing body of literature which suggests there is a need to develop triage tools to more accurately triage injured children and older adults [27, 42]. While we decided to exclude older adults and pediatric populations from the current review, age was included as a variable in several included TTTs, and often these age groups are included in TTTs in clinical practice. This can be seen as a limitation of our approach and future work is needed to address this.

In developing the search strategy, we ran exploratory searches and found the use of the terms ‘assessment’ or ‘evaluation’ led to too broad a search, with many thousands of extra irrelevant hits, which we did not have the resources to review. This may have resulted in the omission of potentially relevant articles. However, given our hybrid approach of searching for systematic reviews, supplemented with the search for individual studies from more recent literature, it was possible to identify a substantial number of articles to be included in the review (*n* = 92). Therefore, it is unlikely that important articles will have been omitted.

It was not possible for us to include non-English language publications. Potentially, this may have resulted in the omission of some non-English tools. However, we did find a range of tools from non-English speaking countries (such as Denmark, Italy, Norway, Sweden, France, the Netherlands and Norway) which were translated and published in English (see Appendix 3).

Finally, tools that did not apply standardized approach to assessment were excluded. Potentially, this could have resulted in the omission of tools based on modern technology that have yet to establish a standardized approach to assessment. Tools without a standardized approaches to assessment were excluded as the review was conducted to inform the development of a trauma triage tool that could be used by prehospital staff to identify major trauma patients. As such a tool would be used by practitioners in a range of contexts, it is important that it would apply standardized approaches for assessment. Should the application of such a tool not involve a standardized approach, there is a potential that this could expose patients to sub-optimal clinical decision-making. These could be explored in future research.

### Future research

The findings of the review suggest a need to develop an agreed taxonomy of the approach to the measurement of prehospital characteristics of major trauma patients. This would consolidate measures to enable a more concise and consistent identification of the interaction between such characteristics and how these characteristics support the identification of major trauma patients. From an international perspective, we may find slight variation in some categories applied to a TTT according to the population characteristics, for example more focus on falls in areas with a higher proportion of older patients. Nonetheless, an agreed taxonomy could avoid researchers developing further TTTs with multiple yet slightly differing thresholds for variables.

In tandem with this, it would be appropriate to apply greater degrees of transparency in the publication of trauma triage tools. That is, publishing protocols, making datasets used in the analysis publicly available and sharing the codes developed for the analysis. This follows the recommendations of White and colleagues [19].

## Conclusion

This review found that while there is similarity in the higher-level categories included in TTTs - physiological characteristics, injury characteristics, mechanism of injury and any modifiers for high-risk groups– there is very considerable heterogeneity in the specific variables included and their operationalization internationally. The extensive heterogeneity in the thresholds applied TTTs suggests an urgent need for uniform adoption of agreed operationalization’s, along with a need to be more critical and transparent during the process of developing such tools, to prevent sub-optimal clinical decision-making in major trauma triaging.

### Reporting guidelines

We have reported the conduct of this review in accordance with the Preferred Reporting Items for Systematic Review and Meta-Analysis (PRISMA) updated guidelines for reporting systematic reviews [24]. The completed PRISMA Checklist can be found in Appendix 1.

## Electronic supplementary material

Below is the link to the electronic supplementary material.

Supplementary Material 1

Supplementary Material 2

Supplementary Material 3

Supplementary Material 4

Supplementary Material 5

## Data Availability

Data is provided within the manuscript or supplementary information files.
