# Supplementary material for: Substantial heterogeneity in trauma triage tool characteristic operationalization for identification of major trauma: a hybrid systematic review
Source: Eur J Trauma Emerg Surg. 2025 Jan 24;51(1):74. doi: 10.1007/s00068-024-02694-6 (PMC11842439; doi:10.1007/s00068-024-02694-6)
Supplement: Supplementary file 1 — Supplementary Material 1 [file 68_2024_2694_MOESM1_ESM.docx]

# Appendix 1: PRISMA Checklist

| Section and topic | Item # | Checklist item | Reported on page |
| --- | --- | --- | --- |
| **Title** |  |  |  |
| Title | 1 | Prehospital characteristics that identify major trauma patients: a hybrid systematic review | 1 |
| **Abstract** |  |  |  |
| Abstract | 2 | **Background:** Trauma Triage Tools (TTTs) support paramedic staff to identify major trauma patients based on prehospital characteristics and bring them to appropriate trauma centres. However, while triaging trauma has been examined extensively, there appears to be no consensus on the minimum criteria for prehospital identification of major trauma or how variables within TTTs are operationalised.  **Objective:** Examine the prehospital characteristics and their operationalisation applied in the international literature in TTTs.  **Methods:** We applied a hybrid review approach in accordance with best practice guidelines. Searches were conducted in Pubmed (Ovid MEDLINE), Embase, Cochrane Library of Systematic Reviews and Cochrane Central Register of Clinical Trials. We searched for systematic reviews that analyse prehospital characteristics applied in TTTs. This search was supplemented with an updated search of original TTT papers from November 2019. We conducted duplicate screening of all articles by two reviewers and a third reviewer was employed to arbitrate disputes. Data was extracted using a pre-defined data extraction template.  **Results:** We identified 92 papers from which 52 adult general population TTTs were found. The study results suggest that there is considerable heterogeneity in prehospital characteristics included in trauma triage tools internationally. We found congruity in the higher-level categories included in the tools. That is, tools often included measurements of a patient’s physiological characteristics, injury characteristics, mechanism of injury and any modifiers for high-risk groups. However, the prehospital characteristics that make up those groups, how they are categorised and how they are interpreted were found to vary considerably between the tools identified.  **Conclusions:** While there is agreement in the higher-level categories used in TTTs, the thresholds adopted in specific variables varies widely. This may contribute to considerable variation in standards of major trauma triaging internationally. The results suggest a need to develop an agreed taxonomy of the approach to operationalise prehospital characteristics of major trauma patients and a need to be more critical and transparent during the process of developing such tools, to prevent sub-optimal clinical decision-making in major trauma triaging. | 1-2 |
| **Introduction** |  |  |  |
| Rationale | 3 | there is no consensus on an optimal triage tool (15) and with that, no consensus on the minimum criteria for prehospital identification of major trauma (20). Examining and synthesizing the prehospital characteristics applied to identify major trauma patients could better inform the development of future trauma triage tools by identifying best practice for same. We therefore aimed to document the range of variables included in TTTs and explore the heterogeneity of the adopted pre-hospital characteristics in terms of their operationalisation. | 3-4 |
| Objectives | 4 | Examine the prehospital characteristics and their operationalisation applied in the international literature in TTTs. | 4 |
| **Methods** |  |  |  |
| Eligibility criteria | 5 | **Studies:** Studies that were eligible to be included in the review were papers on TTTs that aimed to identify major trauma patients. Only English language papers were included. We excluded studies of triage outside of the prehospital setting, studies concerned with mass casualty trauma events, in-hospital trauma team response, and studies only concerned with activation of helicopter response.  **Participants:** We included studies on TTTs conducted for the general adult population. Patients with medical needs that are not the direct result of an injury were excluded. Studies which were confined to a specific cohort of trauma patients, namely paediatric and older patient populations, were excluded.  **Variables of interest:** Prehospital characteristics applied in trauma triage tools that are associated with major trauma. Medical needs that are not the direct result of an injury (e.g., diabetes) were excluded. Triage tools that did not apply a standardised approach to assess prehospital characteristics, or if the characteristics were not described in the tool, were excluded. | 4-5 |
| Information sources | 6 | Ovid MEDLINE, Embase, Cochrane Library of Systematic Reviews and Cochrane Central Register of Clinical Trials were searched on the 31^st^ January 2023. | 6 |
| Search strategy | 7 | The database search included a search of subject headings, sub-headings, keywords, concept words and associated synonyms. The search terms were: Trauma, Trauma Centres, trauma system; triage, under-triage, over-triage; systematic review, meta-analysis.  **Example of Medline (Ovid) search Strategy**  1 Trauma.mp. or exp Trauma Centers/ or (trauma adj1 centre$).mp. or (trauma adj1 center$).mp. or (trauma adj1 system$).mp.  2 exp Triage/ or triage.mp. or undertriage.mp. or overtriage.mp.  3 1 AND 2  4 ((systematic or scoping or literature) adj (review* or overview*)).mp  5 ("review* of reviews" or meta-analy* or metaanaly* or metasynthe* or meta-synthe*).mp.  6 exp Review Literature as Topic/ or exp Review/ or Meta-Analysis as Topic/ or Meta-Analysis/ or "systematic review"/  7 4 OR 5 OR 6  8 3 AND 7 | 6 |
| Selection process | 8 | Two reviewers (ML and RZ) undertook duplicate screening of titles and abstracts of papers identified by the literature search. Papers that do not meet the inclusion criteria were excluded. Disagreements were discussed with a third reviewer (ND). | 6 |
| Data collection process | 9 | A pre-defined data extraction template was applied. | 6 |
| Data items | 10 | Extracted data included the study author(s), year of publication, country, name of the triage tool or protocol, study population, sample size, study design, measures of major trauma applied in the study and prehospital characteristics used to identify major trauma patients. | 6 |
| Synthesis methods | 11a | The objective of the review was to examine the prehospital characteristics applied in the international literature to identify major trauma patients. In particular, to comprehensively identify the prehospital characteristics and the thresholds applied in those tools that identify major trauma patients. Therefore, narrative synthesis was the most appropriate method to analyse the results. | 7 |
|  | 11b | The results have been presented using a traffic light system. That is, where 60% or more of the tools included a variable, this is presented in green. Where 30-59% of the tools included the variable, this is presented in orange. If less than 30% of the tools included the variable, this is presented in in red. | 7 |
| **Results** |  |  |  |
| Study selection | 12 | Starting with the search for systematic reviews. The database search identified 1,144 records. There were 223 duplicates which were removed. Following abstract screening, 886 articles were excluded. Thirty-five full text articles were assessed for eligibility, from which 29 were excluded (15 did not identify trauma patients, 7 were not systematic reviews, 2 were specific to paediatric populations and 5 were specific to older patient populations). Thus, 70 individual papers from 6 systematic reviews were included in the analysis.  The database search for original articles, post November 2019, identified 2,242 records. From these records, 664 duplicates were removed. Abstract screening identified 1,523 records which did not meet the inclusion criteria and so were removed. A further 55 full text articles were assessed for eligibility, from which 33 articles were excluded (11 did not identify trauma patients and 22 were not in a prehospital setting). This left 22 full text articles to be included in the analysis. Therefore, between the systematic reviews which identified 70 individual papers and the 22 original articles, data from a total of 92 studies were included in the analysis. | 7 |
| Study characteristics | 13 | See Appendix 3 |  |
| Results of syntheses | 14 | Tables 1-4 below present the synthesised results of the prehospital characteristics included in the 52 identified trauma triage tools. There appears to be a broad overlap in the higher-level categories of prehospital characteristics included in TTTs. It would appear that most TTTs account for a patient’s physiological characteristics, injury characteristics, the mechanism of injury and then modifiers for high-risk groups. However, there is considerable variation between TTTs in the make-up and operationalisation of these groups and individual variables. | 10-16 |
| **Discussion** |  |  |  |
| Discussion | 15 | This is the first review to quantify the most commonly used variables in TTTs and highlight the heterogeneity of variable operationalisation within these. We identified 92 different articles and 52 trauma triage tools. Such a wide range of articles and trauma triage tools meant we could comprehensively establish the range of prehospital characteristics that have been applied to identify major trauma patients. The study results suggest that there is considerable heterogeneity in prehospital characteristics included in trauma triage tools internationally. We found congruity in the higher-level categories included in the tools. That is, tools often included measurements of a patient’s physiological characteristics, injury characteristics, mechanism of injury and any modifiers for high-risk groups. However, the prehospital characteristics that make up those categories, how they are categorised and how they are interpreted were found to vary considerably between the tools identified. Given the substantial differences found between the tools, especially thresholds included, the results suggest considerable variation in standards of major trauma triaging internationally. | 16-17 |
|  | 16 | Discuss any limitations of the evidence included in the review. |  |
|  | 17 | In developing the search strategy, we ran exploratory searches and found the use of the terms ‘assessment’ or ‘evaluation’ led to too broad a search, with many thousands of extra irrelevant hits, which we did not have the resources to countenance. This may have resulted in the omission of potentially relevant articles. However, given our hybrid approach of searching for systematic reviews, supplemented with the search for individual studies from more recent literature, it was possible to identify a substantial number of articles to be included in the review (n=92). Therefore, it is unlikely that important articles will have been omitted.  Given resource constraints, it was not possible for us to include non-English language publications. Potentially, this may have resulted in the omission of some non-English tools. However, we did find a range of tools from non-English speaking countries (such as Denmark, Italy, Norway, Sweden, France, the Netherlands and Norway) which were translated and published in English (see Appendix 3). Thus, we do not anticipate the omission of non-English language publications would significantly impact study findings.  Finally, tools that did not apply standardised approach to assessment were excluded. Potentially, this could have resulted in the omission of tools based on modern technology that have yet to establish a standardised approach to assessment. Tools without a-standardised approaches to assessment were excluded as the review was conducted to inform the development of a trauma triage tool that could be used by paramedics to identify major trauma patients. As such a tool would be used by practitioners in a range of contexts, it is important that it would apply standardised approaches for assessment. Should the application of such a tool not involve a standardised approach, there is a potential that this could expose patients to sub-optimal clinical decision-making. These could be explored in further research in the future. | 18-19 |
|  | 18 | The findings of the review suggest a need to develop an agreed taxonomy of the approach to the measurement of prehospital characteristics of major trauma patients. This would consolidate measures to enable a more concise and consistent identification of the interaction between such characteristics and how these characteristics support the identification of major trauma patients. From an international perspective, we may find slight variation in some categories applied to a TTT according to the population characteristics, for example more focus on falls in areas with a higher proportion of older patients. Nonetheless, an agreed taxonomy could avoid researchers developing further TTTs with multiple yet slightly differing thresholds for variables.  In tandem with this, it would be appropriate to apply the greater degrees of transparency in the publication of trauma triage tools. That is, publishing protocols, making datasets used in the analysis publicly available and sharing the codes developed for the analysis. This follows the recommendations of White and colleagues (22). | 19 |
| **Other information** | |  |  |
| Registration and protocol | 19 | This hybrid review was registered with the International Prospective Register of Systematic Reviews (PROSPERO) database (Protocol Number: CRD42023393094). | 20 |
|  | 20 | The protocol for the review was published in HRB Open (44). | 20 |
|  | 21 | There were a number of deviations to information provided in the protocol. Resource and time constraints meant it was not possible to assess the quality of studies included in the review. As the objective of the review was examine the prehospital characteristics applied in the international literature, we needed as extensive a range of tools included as was possible. The quality of the studies would not have impacted the study findings given it was important that as many prehospital characteristics from triage tools were included, irrespective of the quality of the tools.  Secondly, the protocol specified that studies confined to a specific population (paediatrics or older patient populations) would be included in the review and analysed by separating out those tools that are specific to populations. However, it was subsequently decided to exclude studies which were confined to paediatric and older patient populations. There were a number of reasons for this. Firstly, as the review was conducted to inform the development of a clinical prediction tool for the general trauma patient population, it was important that the evidence informing the development of the clinical prediction tool was appropriate to the patient population to which it would be applied. In tandem with this, paediatric and older patient populations have differing physiological characteristics, for example the general systolic blood pressure and respiratory rate for young children and adults over the age of 65 are considerably different (28-32). These groups also often present with specific and complex care needs. Thus, there is a considerable and growing body of literature to suggest trauma triaging in paediatric or older adult populations should include criteria that is more sensitive to standard adult criteria in identifying major trauma patients that would benefit from care in a major trauma centre (28-32).  Finally, we had hoped to examine alternative definitions of major trauma in the analysis. However, following data extraction it became apparent that this would not be possible given that the majority of studies applied the Injury Severity Score (ISS) to define major trauma patients. The Trauma and Injury Severity Score (TRISS) (36) was only applied in one study and the New Injury Severity Score (NISS) (37) was only applied in two studies. | 20-21 |
| Support | 22 | This research is funded by the Health Research Board (HRB) under Grant Number SDAP-2021-006. | 21 |
| Competing interests | 23 | None | 21 |
| Availability of data, code, and other materials | 24 | The data used for all analyses can be found in Appendix 4. | |
