# Supplementary material for: Substantial heterogeneity in trauma triage tool characteristic operationalization for identification of major trauma: a hybrid systematic review
Source: Eur J Trauma Emerg Surg. 2025 Jan 24;51(1):74. doi: 10.1007/s00068-024-02694-6 (PMC11842439; doi:10.1007/s00068-024-02694-6)
Supplement: Supplementary file 2 — Supplementary Material 2 [file 68_2024_2694_MOESM2_ESM.docx]

**Prehospital characteristics that identify major trauma patients: A hybrid systematic review. Donnelly et al.,**

**Appendix 2 Search Strategy at 31 Jan 2023**

**Search 1 – Search for Systematic Reviews of Trauma Triage Tools**

|  | OVID MEDLINE all | 31 Jan |
| --- | --- | --- |
|  |  |  |
| 1 | Trauma.mp. or exp Trauma Centers/ or (trauma adj1 centre$).mp. or (trauma adj1 center$).mp. or (trauma adj1 system$).mp. | 311576 |
| 2 | exp Triage/ or triage.mp. or undertriage.mp. or overtriage.mp. | 29559 |
| 3 | 1 AND 2 | 4109 |
| 4 | ((systematic or scoping or literature) adj (review* or overview*)).mp. | 433636 |
| 5 | ("review* of reviews" or meta-analy* or metaanaly* or metasynthe* or meta-synthe*).mp. | 296152 |
| 6 | exp Review Literature as Topic/ or exp Review/ or Meta-Analysis as Topic/ or Meta-Analysis/ or "systematic review"/ | 3305924 |
| 7 | 4 or 5 or 6 | 3424636 |
| 8 | 3 and 7 | 561 |
|  |  |  |
|  | EMBASE |  |
| 1 | trauma:ti,ab,kw OR ((trauma NEXT/1 center$):ti,ab,de,kw) OR ((trauma NEXT/1 centre$):ti,ab,de,kw) OR ((trauma NEXT/1 system$):ti,ab,de,kw) | 372,074 |
| 2 | 'patient triage'/exp OR triage:ti,ab,de,kw OR undertriage:ti,ab,de,kw OR overtriage:ti,ab,de,kw | 37,877 |
| 3 | #1 AND #2 | 4,203 |
| 4 | ((systematic OR scoping OR literature) NEXT/1 (review$ OR overview$)):ti,ab,de,kw | 644,126 |
| 5 | "review$ of reviews":ti,ab,de,kw OR 'meta analy*':ti,ab,de,kw OR metaanaly*:ti,ab,de,kw OR metasynthe*:ti,ab,de,kw OR 'meta-synthe*':ti,ab,de,kw | 420,752 |
| 6 | 'literature'/exp OR ‘review’/exp OR 'meta analysis (topic)'/exp OR 'meta analysis'/exp OR ‘systematic review’/exp | 3,446,989 |
| 7 | #4 OR #5 OR #6 | 3,634,126 |
| 8 | #3 AND #7 | 451 |
|  |  |  |
|  | COCHRANE LIBRARY OF SYSTEMATIC REVIEWS & CENTRAL REGISTER OF CLINICAL TRIALS |  |
| 1 | Trauma:ti,ab,kw OR (trauma NEAR/1 center$):ti,ab,kw OR (trauma NEAR/1 centre$):ti,ab,kw OR (trauma NEAR/1 system$):ti,ab,kw | 18120 |
| 2 | (triage or undertriage or overtriage):ti,ab,kw | 1963 |
| 3 | 1 AND 2 | 132 |
|  |  |  |

**Search 2 – Search for original papers examining Trauma Triage Tools since Nov 2019**

|  | OVID MEDLINE all | 31 Jan |
| --- | --- | --- |
|  |  |  |
| 1 | Trauma.mp. or exp Trauma Centers/ or (trauma adj1 centre$).mp. or (trauma adj1 center$).mp. or (trauma adj1 system$).mp. | 311576 |
| 2 | exp Triage/ or triage.mp. or undertriage.mp. or overtriage.mp. | 29559 |
| 3 | 1 AND 2 | 4109 |
| 4 | limit 3 to dt=20191101-20230131 | 860 |
| 5 | limit 3 to ed=20191101-20230131 | 810 |
| 6 | 4 or 5 | 1025 |
|  |  |  |
|  | EMBASE |  |
| 1 | trauma:ti,ab,kw OR ((trauma NEXT/1 center$):ti,ab,de,kw) OR ((trauma NEXT/1 centre$):ti,ab,de,kw) OR ((trauma NEXT/1 system$):ti,ab,de,kw) | 372,074 |
| 2 | 'patient triage'/exp OR triage:ti,ab,de,kw OR undertriage:ti,ab,de,kw OR overtriage:ti,ab,de,kw | 37,877 |
| 3 | #1 AND #2 | 4,203 |
| 4 | #1 AND #2 AND [01-11-2019]/sd NOT [01-02-2023]/sd | 1,188 |
|  |  |  |
|  | COCHRANE LIBRARY OF SYSTEMATIC REVIEWS & CENTRAL REGISTER OF CLINICAL TRIALS |  |
| 1 | Trauma:ti,ab,kw OR (trauma NEAR/1 center$):ti,ab,kw OR (trauma NEAR/1 centre$):ti,ab,kw OR (trauma NEAR/1 system$):ti,ab,kw | 18120 |
| 2 | (triage or undertriage or overtriage):ti,ab,kw | 1963 |
| 3 | 1 AND 2 | 132 |
| 4 | 3 with Publication Year from 2019 to present , with Cochrane Library publication date from Nov 2019 to present, in Trials | 29 |
