# Supplementary material for: Substantial heterogeneity in trauma triage tool characteristic operationalization for identification of major trauma: a hybrid systematic review
Source: Eur J Trauma Emerg Surg. 2025 Jan 24;51(1):74. doi: 10.1007/s00068-024-02694-6 (PMC11842439; doi:10.1007/s00068-024-02694-6)
Supplement: Supplementary file 3 — Supplementary Material 3 [file 68_2024_2694_MOESM3_ESM.docx]

| **Appendix 3: Characteristics of included studies** | | | | | | | | |
| --- | --- | --- | --- | --- | --- | --- | --- | --- |
| **Data from Systematic Reviews** | | | | | | | | |
| **Systematic Reviews Information** | | | **Data on articles and tools included in the review** | | | | | |
| **Systematic Review First Author** | **Year** | **Title** | **First Author; country; publication year** | **Triage Tool** | **Population** | **Sample Size** | **Study Design** | **Definition of severely injured patient** |
| Gianola | 2021 | Accuracy of pre-hospital triage tools for major trauma: A systematic review with meta-analysis and net clinical benefit | Dinh (1); Australia; 2012 | Ambulance Service of New South Wales- Major Trauma Criteria (MIST) (1) | All adult (>15) years old patients transported by the Ambulance Service of New South Wales | 2,664 | Retrospective | ISS >15 and major outcome (composite of in-hospital death and/or transferred from the ED to operating theatre or intensive care unit) |
|  |  |  | Ocak (2); the Netherlands; 2009 | The American College of Surgeons Committee on Trauma (ACS-COT)(3) | Patients treated at a Dutch trauma centre | 302 | Retrospective | ISS >15 |
|  |  |  |  | New prehospital items not included in ACS-COT triage guidelines (2) | Patients treated at a Dutch trauma centre | 302 | Retrospective | ISS >15 |
|  |  |  | Bouzat (4); France; 2015 | French Vittel Triage Criteria (5) | Patients with suspected severe trauma | 2,572 | Retrospective | ISS >15 |
|  |  |  |  | New TRENAU Grading system (4) | Patients with suspected severe trauma | 2,572 | Retrospective | ISS >15 |
|  |  |  | Bouzat (6); France; 2016 | the Triage-Revised Trauma Score (T-RTS) (7) | Patients with suspected severe trauma according to the Vittel criteria | 3,260 | Retrospective | Death from any cause during hospital stay |
|  |  |  |  | Mechanism Glasgow Age and arterial Pressure (MGAP) score. MGAP (8) | Patients with suspected severe trauma according to the Vittel criteria | 3,260 | Retrospective | Death from any cause during hospital stay |
|  |  |  | Voskens (9); the Netherlands; 2018 | Dutch Field Triage protocol, based on the American College of Surgeons Committee on Trauma (ACS-COT)  (10) | Adult trauma patients | 4,950 | Prospective | ISS >15 |
|  |  |  | van Laarhoven (11); the Netherlands; 2014 | Dutch Field Triage protocol developed by the LPA (National Protocol of Ambulance Services) (10). | High energy trauma patients | 1,607 | Retrospective | ISS >15 |
|  |  |  | Vinjevoll (12); Norway; 2018 | New trauma team activation tool for central Norway trauma system (12) | All patients received by trauma teams in 7 hospitals in Central Norway | 998 | Retrospective | ISS >15 |
|  |  |  | Follin (13); France; 2016 | Triage recommendations from the French Society of Emergency Medicine (14) | All consecutive patients admitted for trauma at a major trauma centre in Paris | 1,160 | Prospective | ISS>15, Spinal Cord Injury, Advanced Airway Management, Emergency Surgery, or In-Hospital Death. |
|  |  |  | Sewalt (15); United Kingdom; 2019 | 1. Circulation, Respiration, Abdomen, Motor, Speech (CRAMS) (16) | Injured patients ages over 15 years who were transported by the emergency medical services or helicopter emergency medical services and admitted to hospital in England between 2013-2016 were included. | 154,476 | Retrospective | ISS >15 and in-hospital mortality |
|  |  |  |  | 2. Prehospital Index (PHI) (17) |  |  |  |  |
|  |  |  |  | 3. Triage Revised Trauma Score (T-RTS) (7) |  |  |  |  |
|  |  |  |  | 4. Physiologic Severity Score (PSS) (18) |  |  |  |  |
|  |  |  |  | 5. Mechanism Glasgow Coma Scale, Age and Arterial Pressure (MGAP) (8) |  |  |  |  |
|  |  |  |  | 6. the modified Rapid Emergency Medicine Score (mREMS) (19) |  |  |  |  |
|  |  |  |  | 7. Kampala Trauma Score (KTS) (20) |  |  |  |  |
|  |  |  | Cassignol (21); France; 2019 | Vittel field triage criteria (5) | Trauma patients included in the local trauma registry with severe trauma suspected in the pre-hospital setting according to the Vittel field triage criteria | 1,151 | Retrospective | ISS>15, mortality within 30days, ICU admission. |
|  |  |  | Llompart-Pou (22); Spain; 2017 | Mechanism Glasgow Coma Scale, Age and Arterial Pressure (MGAP) (8) | Trauma patient admitted to ICU | 1,361 | Retrospective | Mortality and Trauma and Injury Severity Score (TRISS) |
|  |  |  |  | Glasgow coma scale, age, systolic blood pressure (GAP) (23). | Trauma patient admitted to ICU | 1,361 | Retrospective | Mortality and Trauma and Injury Severity Score (TRISS) |
|  |  |  |  | Triage Revised Trauma Score (T-RTS) (7) | Trauma patient admitted to ICU | 1,361 | Retrospective | Mortality and Trauma and Injury Severity Score (TRISS) |
|  |  |  | Baxt (24); USA; 1990 | Trauma Triage Rule (24) | >14 years admitted to University of California, San Diego Medical Centre trauma centre by paramedic land ambulance | 1,004 | Prospective | SBP<85 mmHg, motor (aspect of GCS) <5, or penetrating trauma of head, neck, or trunk |
| Van Rein | 2017 | Accuracy of prehospital triage protocols in selecting severely injured patients: A systematic review | Bond (25) ; Canada; 1997 | Prehospital Index (17). | >14 years adult trauma patients brought to any adult hospital by City of Calgary EMS | 3,147 | Retrospective | ISS >15 |
|  |  |  |  | Mechanism of Injury combined with Prehospital Index (25). |  |  |  |  |
|  |  |  | Brown (26); USA; 2011 | 2006 Field Triage Decision Scheme by The American College of Surgeons Committee on Trauma (ACS-COT) (27) | Trauma patients >=18 years admitted, transferred or deceased. | 1,086,764 | Retrospective | ISS >15, ICU admission within 24 h, or urgent surgery |
|  |  |  | Champion (7); USA; 1989 | Trauma Score (7) | Patients' physiological data collected from Washington Hospital Centre Database | 2,166 | Retrospective | ISS >15 |
|  |  |  |  | Triage- Revised Trauma Score (7) |  |  |  |  |
|  |  |  | Ciesla (28); USA; 2015 | Field Triage of Florida (28) | Florida Department of Health 2012 Trauma Registry inclusion criteria- all patients who were hospitalised for trauma, regardless of diagnosis. | 116,990 | Retrospective analysis | ICISS <0.85 (International Classification Injury Severity Score) |
|  |  |  | Dihn (29); Australia; 2014 | New South Wales Ambulance Service Major Trauma Transport protocol (29) | Ambulance presentations requiring trauma team activation between 2007 and 2011 in a single inner-city major trauma centre | 3,027 | Retrospective analysis | ISS >15, ICU admission, or in-hospital death |
|  |  |  | Gray (30); United Kingdom; 1997 | Circulation, Respiration, Abdomen, Motor, and Speech (CRAMS) (16) | Patients in the resuscitation room of Leeds General Infirmary with sufficient data to calculate CRAMS/TRTS scores | 213 | Retrospective | ISS >15, ICU admission, or death |
|  |  |  |  | Triage Revised Trauma Score (7) |  |  |  |  |
|  |  |  | Hamada (5); France; 2014 | Vittel Triage Criteria (5) | All patients admitted to the trauma ward of a Paris Hospital, as well as from an electronic database of physician-staffed ambulance data | 825 | Retrospective | ISS>15 |
|  |  |  | Hedges (31); USA; 1987 | Kane's "Revised" Trauma Checklist (32) | Trauma patients transported by ambulance to ED | 130 | Retrospective | No vital signs, death in ED, non-orthopaedic surgery, or ICU admittance |
|  |  |  |  | Circulation, Respiration, Abdomen, Motor, and Speech (CRAMS) (16) |  |  |  |  |
|  |  |  |  | Trauma Score (31) |  |  |  |  |
|  |  |  |  | RSG score (Respiratory, systolic pressure, GCS score) (31) |  |  |  |  |
|  |  |  |  | Prehospital Index (17) |  |  |  |  |
|  |  |  |  | RPM score (Respiratory/pulse/motor response score) (33) |  |  |  |  |
|  |  |  |  | Respiratory/systolic blood pressure/motor response Score (7) |  |  |  |  |
|  |  |  |  | Paramedic severity impression score (34) |  |  |  |  |
|  |  |  | Knopp (35); USA; 1988 | Trauma score (35) | All calls to Fresno county EMS communications centres requesting prehospital care involving traumatic injuries, or transfers from surrounding counties or 3 national parks | 1,473 | Prospective | ISS >15 |
|  |  |  |  | Trauma score with added physiological and anatomical criteria (35) |  |  |  |  |
|  |  |  | Lerner (36); USA; 2011 | 1999 Field Triage Decision Scheme by ACS-COT (3) | All patients brought by ambulance to a regional trauma centre in 3 mid-size cities | 11,891 | Retrospective | ISS >15 |
|  |  |  |  | 2006 Field Triage Decision Scheme by ACS-COT (27) |  |  |  |  |
|  |  |  | Matsushima (37); USA; 2016 | Unnamed motor vehicle intrusion protocol (37) | Trauma patients involved in motor vehicle crash | 3,998 | Retrospective | Intubation at ED, non-orthopaedic surgery, ICU admission, or in-hospital mortality |
|  |  |  | Morris (38); USA; 1986 | Trauma Score (38) | Trauma patients brought by ambulance to San Francisco hospital. | 1,099 | Prospective | ISS>20 |
|  |  |  | Newgard (39); USA; 2016 | 2006 Field Triage Decision Scheme by ACS-COT (27) | Enrolled injured children and adults transported by EMS to an acute care hospital | 17,633 | Prospective | ISS >15 |
|  |  |  | Sturms (40); the Netherlands; 2006 | Triage revised Trauma Score (7) | Major trauma patients treated by the Trauma Centre West-Netherlands and seven non-trauma-centre hospitals | 451 | Retrospective analysis | ISS >15 |
|  |  |  | Tamim (41); Canada; 2002 | Prehospital Index (17) | Trauma patients above 15 years of age, transferred from the injury site to the level 1 trauma centres | 1,291 | Retrospective | Death <8 days, non-orthopaedic surgery <4 days, or ICU admission <7 days |
|  |  |  |  | New Triage Protocol (41) |  |  |  |  |
|  |  |  | Zimmer-Gembeck (42); USA; 1995 | Triage Criteria by Oregon (42) | All admissions for acute injuries in a 2.5 year period | 26,025 | Retrospective | Non-orthopaedic surgery, fluid resuscitation, invasive CNS monitoring, or death |
|  |  |  | Gage (43); USA; 2012 | 2006 Field Triage Decision Scheme by The American College of Surgeons Committee on Trauma (ACS-COT) (27) | Patients aged 18 or older transported by ambulance | 12,106 | Retrospective | ISS >15 |
|  |  |  | Newgard (44); USA; 2017 | 2011 Field Triage Decision Scheme by ASC-COT (45) | Patients transported by EMS in areas study was active in | 17,633 | Pre-planned Secondary Analysis of multi-site, prospective cohort study | ISS >15 |
|  |  |  | Newgard (46); USA; 2011 | 2006 Field Triage Decision Scheme by ACS-COT (27) | Patients of all ages brought to a hospital by ambulance in 7 regions in western USA. | 122,345 | Retrospective | ISS >15 |
| Lupton | 2022 | Under-Triage and Over-Triage Using the Field Triage Guidelines for Injured Patients: A Systematic Review | Scheetz (47); USA; 2011 | 2006 Field Triage Decision Scheme by ACS-COT (27) | Patients >=55 years with a maximum injury severity of uninjured or an Abbreviated Injury Scale score of 1 to 5. | 556,898 | Retrospective | mAIS (maximum Abbreviated Injury Scale) 3-5 |
|  |  |  | Scheetz (48); USA; 2012 | 2006 Field Triage Decision Scheme by ACS-COT (27) | Patients age 65 years or older with a maximum Abbreviated Injury Scale score of 3/ 4 / 5 and transported from the scene of the crash to a trauma centre or non-trauma centre hospital ED | 66,445 | Retrospective | mAIS (maximum Abbreviated Injury Scale) 3-5 |
|  |  |  | Newgard (49); USA; 2013 | 2011 Field Decision Scheme by AS-COT (45) | Patients transported by EMS after a 911 call in 7 geographic areas | 301,214 | Retrospective | ISS >15 |
|  |  |  | Nijishima (50); USA; 2018 | 2011 Field Triage Decision Scheme by ACS-COT and CDC (steps 1-3) (45) | Adults >=55 yrs with head trauma transported to hospital | 1,304 | Prospective | traumatic intracranial haemorrhage |
|  |  |  |  | Sacramento County Trauma Triage Tool. (51) | Adults >=55 yrs with head trauma transported to hospital | 1,304 | Prospective | traumatic intracranial haemorrhage |
|  |  |  | Nijishima (52); USA; 2017 | 2011 Field Triage Decision Scheme by ASC-COT (steps 1-3) (45) | Patients aged 55 years and older with head trauma transported to a hospital by the participating EMS agencies | 2,110 | Retrospective | Intracranial haemorrhage |
|  |  |  | Hon (53); USA; 2020 | 2011 Field Triage Decision Scheme by ASC-COT (45) | Patients 55 years, who experienced blunt head trauma and were transported by EMS | 673 | Prospective | Intracranial haemorrhage |
|  |  |  | Parikh (54); USA; 2017 | 2011 Field Triage Decision Scheme by ASC-COT (45) | Records from state EMS reporting system integrated with trauma registry data | 4,757 | Retrospective | ISS >15 |
|  |  |  |  | State of Ohio guidelines (55) |  |  |  |  |
|  |  |  | Flottemesch (56); USA; 2016 | 2006 Field Triage Decision Scheme by ACS-COT (27) | Patients aged 18 years and older with a head injury treated at an ED with a TC or non-TC designation | 140,766 | Pre-post design | Severe head trauma with an Abbreviated Injury Scale (AIS) score of 4 or higher |
|  |  |  |  | 2011 Field Triage Decision Scheme by ACS-COT (45) |  |  |  |  |
|  |  |  | Barnett (57); USA; 2013 | 2011 Field Triage Decision Scheme by ACS-COT (45) | Injured children and adults evaluated and brought by ambulance to hospitals in 6 geographical regions in the western USA | 46,414 | Retrospective | Not detailed |
|  |  |  | Davidson (58); USA; 2014 | 2012 CDC Guidelines for Field Triage of Injured Patients (45) | National Automotive Sampling System Crashworthiness Data System 2003 to 2008 | 85,761 | Retrospective | ISS >15 |
| Bhaumik | 2022 | Prehospital triage tools across the world: a scoping review of the published literature | Cox (59); Australia; 2012 | Victorian state prehospital trauma triage criteria (59) | Trauma patients aged >=16 years old transported by ambulance to a hospital in metropolitan Melbourne | 45,332 | Retrospective | Death, ISS > 15, ICU ventilation or urgent surgery |
|  |  |  | Kann (60); Denmark; 2007 | 1999 Field Triage Decision Scheme by ACS-COT (3), | Patients triaged to Aarhus Trauma centre | 848 | Prospective | ISS>15 |
|  |  |  | Lehmann (61) ; USA; 2007 | Pierce County (WA) prehospital trauma triage guidelines (61) | Trauma patients older than 16 | 1,782 | Retrospective | ISS >15 |
|  |  |  |  | Simplified Triage System (61) |  |  |  |  |
|  |  |  | Lehmann (62) ; USA; 2009 | Pierce County Prehospital Trauma Triage Guidelines (2009) (61) | Trauma patients older than 16 | 244 | Prospective | ISS >15 |
|  |  |  |  | Simplified Triage System (61) |  |  |  |  |
|  |  |  | Haas (63); Canada; 2010 | Does not report a specific protocol | Adult subjects (aged 18 years or older) presenting to any ED with a severe injury | 11,398 | Retrospective | ISS >15 |
|  |  |  | Cotte (64); France; 2016 | French Vittel Criteria (5) | Trauma patients aged >=18 with at least one positive Vittel criterion | 252 | Retrospective | "Vital distress" (at least one of the Vittel criteria related to abnormal prehospital physiological data, need for intensive resuscitation or obvious severe neurological impairment) |
|  |  |  | Shawhan (65); USA; 2015 | Pierce County pre-hospital trauma triage procedures (Shawhan 2015) (61) | Trauma patients aged>=16 years brought to Madigan Army Medical Centre | 704 | Prospective | Requiring a life-saving intervention |
|  |  |  |  | New two-tiered triage protocol (65) |  |  |  |  |
|  |  |  | Davis (66); USA; 2017 | Community Regional Medical Centre tiered trauma activation criteria derived from ACS-COT criteria (66) | Trauma patients at a level 1 trauma centre | 7,031 | Retrospective | ISS >15 |
|  |  |  | Vassallo (67) ; United Kingdom; 2017 | Modified Physiological Triage Tool (68) | Patients in TARN (UK Trauma Audit Research Network) aged >=18yrs admitted directly from site of accident | 218,985 | Retrospective | Priority one (Requiring a life-saving intervention) |
|  |  |  |  | UK Military Sieve (68) |  |  |  |  |
|  |  |  |  | Major Incident Medical Management and Support Triage Sieve (68) |  |  |  |  |
|  |  |  |  | UK National Ambulance Resilience Unit (NARU)(68) |  |  |  |  |
|  |  |  | Báez (69); USA; 2003 | 1999 Field Triage Decision Scheme by ACS-COT (3) | Trauma patients admitted to hospital aged >17 | 37,276 | Cross-sectional | ISS >15 |
| Najafi | 2019 | Determination of mis-triage in trauma patients: a systematic review | Candefjord (70); Sweden; 2016 | Rapid Emergency Trauma and Triage System (RETTS) (71) | Patients involved in road traffic accidents | 117,730 | Retrospective | ISS >15 |
|  |  |  | Ciesla (72); USA; 2017 | Triage criteria of Florida 2010 (28) | Florida Department of Health Trauma Registry inclusion criteria- Patients who were hospitalised for trauma, regardless of diagnosis. | 111,675 | Retrospective analysis | ICISS <0.85 (International Classification Injury Severity Score) |
|  |  |  | Di Bartolomeo (73); Italy; 2004 | Not reported. | Patient admitted with an ISS>15 | 627 | Prospective | ISS>15 |
|  |  |  | Faul (74); USA; 2016 | 2011 Field Triage Decision Scheme by ACS-COT (45) | Patients with traumatic brain injury | 351,555 | Retrospective analysis | Traumatic brain injury, not defined further |
|  |  |  | Garwe (75) ; USA; 2017 | Field Triage Decision Scheme by ACS-COT (45) | Trauma patients with AIS>3, ISS>9, TRISS<0.5 or who died | 49,271 | Retrospective analysis | ISS>15 |
|  |  |  | Haas (76); Canada; 2012 | Not reported. | Trauma patients involved in motor vehicle crash |  | Retrospective analysis | ISS>15 or death within 24 hours of presentation |
|  |  |  | Hsia (77); USA; 2010 | Not reported. | Trauma patients admitted to hospital aged >17 | 752,706 | Retrospective | ISS>15 |
|  |  |  | Kodadek (78); USA; 2015 | 2014 Field Triage Decision Scheme by ACS-COT. (79) | Trauma patients aged >=55 years | 4,152,541 | Retrospective | NISS>8 |
|  |  |  | Kreis (80); USA; 1988 | Trauma Score (81) | Trauma patients brought to hospital by EMS Providers | 8,891 | Prospective | Death in ER, emergency surgery, or ICU admission |
|  |  |  | Lale (82); USA; 2017 | 2011 Field Triage Decision Scheme by ACS-COT (45) | Patients with a firearm-related injury. | 9,886 | Retrospective | NISS>15 (New Injury Severy Score, based on the Abbreviated Injury Sale (83) |
| Van Rein | 2018 | Effectiveness of prehospital trauma triage systems in selecting severely injured patients: Is comparative analysis possible? | Long (84); USA; 1986 | Not reported. | Trauma patients admitted to hospital. | 2,511 | Retrospective | ISS>15 |
|  |  |  | Newgard (85); USA; 2008 | Oregon state trauma triage criteria (42) | Trauma patients that met the triage criteria | 33,699 | Retrospective | ISS>15, Major non-orthopaedic surgery, ventilation >96h, blood transfusion, ICU stay>=2 days or in-hospital mortality. |
|  |  |  | Newgard (86); USA; 2017 | 2006 Field Triage Decision Scheme by ACS-COT (27) | Patients transported by EMS providers | 53,487 | Prospective | ISS>15 or critical resource use |
|  |  |  | Rubenson Wahlin (87); Sweden; 2016 | Triage protocol of Sweden (87) | Trauma patients aged >15 years with an ISS>15 transported by EMS providers | 693 | Retrospective | ISS>15 |
|  |  |  | Santaniello (88); USA; 2003 | 1999 Field Triage Decision Scheme by ACS-COT (3). | Trauma patients aged >17 years | 830 | Retrospective | ISS>15 |
|  |  |  | Scheetz (89); USA; 2004 | Not reported. | Trauma patients aged >24 involved in motor vehicle accident | 5,712 | Retrospective | ISS>15 |
|  |  |  | Tamin (90); Canada; 2002 | Prehospital Index (17) | Trauma patients above 15 years of age, transferred from the injury site to the level 1 trauma centres | 1,291 | Retrospective | Death <8 days, non-orthopaedic surgery <4 days, or ICU admission <7 days |
|  |  |  | Twijnstra (91); the Netherlands; 2010 | Dutch Field Triage protocol developed by the LPA (National Protocol of Ambulance Services) (10) | Trauma patients admitted to hospital in the central region of the Netherlands. | 34,840 | Retrospective | ISS >15 |
|  |  |  | West (92); USA; 1986 | Orange County Triage Criteria (92) | Trauma patients transported by EMS providers | 1,793 | Retrospective | ISS >15 or death |
|  |  |  |  |  |  |  |  |  |

| **Data from post November 19 Original articles** | | | | | | |
| --- | --- | --- | --- | --- | --- | --- |
|  | **First Author; country; publication year** | **Triage Tool** | **Population** | **Sample Size** | **Study Design** | **Definition of severely injured patient** |
|  | Andrews (93); Australia; 2021 | Queensland Ambulance Service's clinical practice guideline: Pre-hospital trauma by-pass. (94) | Road trauma patients | 3,847 | Retrospective | International classification of disease based injury severity score (ICISS) <=0.941 |
|  | Atiksawedparit (95); Thailand; 2019 | Prehospital prediction of severe injury in RTI subjects (95) | Road trauma patients | 2,097 | Prospective | ISS >15 |
|  | Bischoff (96) ; Canada; 2021 | Ontario provincial prehospital trauma triage guidelines (97) | Trauma patients | 460 | Retrospective | Frequency patients met triage standards & which specific criteria were satisfied. |
|  | Bosson (98); USA; 2019 | Los Angeles County Trauma Triage Decision Scheme (99) | Trauma patients aged >15 years | 71,536 | Retrospective | Non-orthopaedic operative intervention within 6 hours of arrival, injury severity score (ISS) > 15, or surgical ICU admission |
|  | Fuller (100); United Kingdom; 2021 | Reference standard defining which patients would benefit from expedited MTC care. (100) | Trauma patients | N/A | Expert and PPI consensus | Need for critical interventions; presence of significant individual anatomical injuries; burden of multiple minor injuries; and important patient attributes |
|  | Hansen (101); Denmark; 2022 | Regional trauma triage guideline (101) | Trauma patients | 11,808 | Retrospective | not defined |
|  | Meyers *(INSERT REF - The Triage of Older Adults with Physiologic Markers of Serious Injury Using a State-Wide Prehospital Plan); USA; 2019 | Trauma Triage Destination Plans (TTDPs) North Carolina (Ref: The North Carolina Office of Emergency Medical Services. NC EMS Triage and Destination Plans 2019 Ref in JM Library) | Trauma patients transported by EMS | 5345 | Retrospective | not defined |
|  | Yeung (102); Hong Kong; 2022 | Prehospital trauma diversion protocol (103) | Trauma patients | 502 | Cross-sectional | not defined |
|  | Bagnato (104); Italy; 2022 | the American College of Surgeons Committee on Trauma (ACS-COT) field triage decision scheme (45) | Trauma patients brought to hospital by EMS Providers | 1,439 | Retrospective | ISS >15 |
|  |  | Northern French Alps (TRENAU) Trauma System Score (4) |  |  |  |  |
|  | Benhamed (105); France; 2022 | Regional triage protocol based on the Vittel Criteria (105) | Trauma patients brought to hospital by EMS Providers | 7,110 | Retrospective | ISS >15 |
|  | Cassignol (106); France; 2019 | Triage Revised Trauma Score (T-RTS) (7) | Trauma patients included in the trauma registry in southern France. | 1,112 | Retrospective | In-hospital mortality |
|  |  | Vittel Criteria (5) |  |  |  |  |
|  |  | Mechanism/Glasgow Coma Scale/Age/Systolic Blood Pressure (MGAP) (8) |  |  |  |  |
|  |  | New Trauma Score (NTS) Jeong (107) |  |  |  |  |
|  | Deeb (108); USA; 2021 | National Field Triage Guidelines (45) | Trauma patients aged 16 and older | 669,795 | Retrospective | Trauma centre need: composite of ISS>15, ICU admission, Urgent surgery, ED death. |
|  | Escobar (109); USA; 2022 | National Field Triage Guidelines (45) | Trauma patients with prehospital acuity of critical or emergent by EMS. | 94,250 | Retrospective | not included |
|  | Fernandez (110); USA; 2023 | The Centres for Disease Control and Prevention Guidelines for Field Triage of Injured Patients (45) | Injured patients excluding children and cardiac arrests | 86,462 | Retrospective | Hospitalisation and emergency department of in-hospital mortality |
|  | Kang (111); USA; 2022 | National Field Triage Guidelines ((45) | Injured patients | 2438 | Retrospective | ISS>15 |
|  | Leichtle (112); USA; 2019 | National Field Triage Guidelines (45) | Trauma patients | 498 | Retrospective | ISS>15 and a composite of hypotension, need for blood transfusions, or immediate operation. |
|  | Mohammed (113); 2022 | Revised Trauma Score (RTS) (114) Mechanism/Glasgow Coma Scale/Age/Pressure (MGAP) score (Sartorius (8) and Glasgow Coma Scale/Age/Pressure (GAP)(23) | Trauma patients | 294 | Retrospective | Mortality |
|  | Moran (115); 2021 | Triage-Revised Trauma Score (T-RTS) (7) | N/A | N/A | Literature review | N/A |
|  | Newgard (116); USA; 2022 | National guideline for the field triage of injured patients: Recommendations of the National Expert Panel on Field Triage (116) | N/A | N/A | Interdisciplinary national expert panel, 5 systematic reviews, EMS feedback and stakeholder feedback | N/A |
|  | Newgard (117); USA; 2019 | 2006 Field Triage Decision Scheme by ACS-COT (27) | Trauma patients | 5021 | Retrospective | ISS>15 or required non-orthopaedic surgery |
|  | Shanahan (118); United Kingdom; 2021 | Van Rein 2019 New prehospital trauma triage protocol (119) | All patients aged >=16 with a suspected injury and transported by ambulance | 68799 | Retrospective | ISS>15 |
|  | Van Rein(119); the Netherlands; 2019 | Van Rein 2019 New prehospital trauma triage protocol (119) | Patients with trauma aged 16+ transported to a trauma centre in 2 regions of the Netherlands | 4,950 | Retrospective | ISS >15 |

1. Dinh MM, Oliver M, Bein KJ, Roncal S, Byrne CM. Performance of the New South Wales Ambulance Service major trauma transport protocol (T1) at an inner city trauma centre. Emergency Medicine Australasia. 2012;24(4):401-7.

2. Ocak G, Sturms LM, Hoogeveen JM, Le Cessie S, Jukema GN. Prehospital identification of major trauma patients. Langenbecks Arch Surg. 2009;394(2):285-92.

3. Committee on the Trauma of the American College of Surgeons. Resources for the Optimal Care of the Injured Patient: 1999. Chicago (IL). American College of Surgeons.; 1999.

4. Bouzat P, Ageron F-X, Brun J, Levrat A, Berthet M, Rancurel E, et al. A regional trauma system to optimize the pre-hospital triage of trauma patients. Critical Care. 2015;19(1):111.

5. Hamada SR, Gauss T, Duchateau FX, Truchot J, Harrois A, Raux M, et al. Evaluation of the performance of French physician-staffed emergency medical service in the triage of major trauma patients. J Trauma Acute Care Surg. 2014;76(6):1476-83.

6. Bouzat P, Legrand R, Gillois P, Ageron FX, Brun J, Savary D, et al. Prediction of intra-hospital mortality after severe trauma: which pre-hospital score is the most accurate? Injury. 2016;47(1):14-8.

7. Champion HR, Sacco WJ, Copes WS, Gann DS, Gennarelli TA, Flanagan ME. A revision of the Trauma Score. J Trauma. 1989;29(5):623-9.

8. Sartorius D, Le Manach Y, David JS, Rancurel E, Smail N, Thicoïpé M, et al. Mechanism, glasgow coma scale, age, and arterial pressure (MGAP): a new simple prehospital triage score to predict mortality in trauma patients. Crit Care Med. 2010;38(3):831-7.

9. Voskens FJ, van Rein EAJ, van der Sluijs R, Houwert RM, Lichtveld RA, Verleisdonk EJ, et al. Accuracy of Prehospital Triage in Selecting Severely Injured Trauma Patients. JAMA Surgery. 2018;153(4):322-7.

10. Nederland Ambulance Zorg. Landelijk Protocol Ambulancezorg (LPA). 2007 2007 [

11. van Laarhoven JJ, Lansink KW, van Heijl M, Lichtveld RA, Leenen LP. Accuracy of the field triage protocol in selecting severely injured patients after high energy trauma. Injury. 2014;45(5):869-73.

12. Vinjevoll OP, Uleberg O, Cole E. Evaluating the ability of a trauma team activation tool to identify severe injury: a multicentre cohort study. Scand J Trauma Resusc Emerg Med. 2018;26(1):63.

13. Follin A, Jacqmin S, Chhor V, Bellenfant F, Robin S, Guinvarc’h A, et al. Tree-based algorithm for prehospital triage of polytrauma patients. Injury. 2016;47(7):1555-61.

14. Riou B, Carli P. Le traumatisé grave. Comment évaluer la gravité. 2002.

15. Sewalt CA, Venema E, Wiegers EJA, Lecky FE, Schuit SCE, den Hartog D, et al. Trauma models to identify major trauma and mortality in the prehospital setting. British Journal of Surgery. 2019;107(4):373-80.

16. Gormican SP. CRAMS scale: field triage of trauma victims. Ann Emerg Med. 1982;11(3):132-5.

17. Koehler JJ, Baer LJ, Malafa SA, Meindertsma MS, Navitskas NR, Huizenga JE. Prehospital Index: a scoring system for field triage of trauma victims. Ann Emerg Med. 1986;15(2):178-82.

18. Husum H, Gilbert M, Wisborg T, Van Heng Y, Murad M. Respiratory rate as a prehospital triage tool in rural trauma. J Trauma. 2003;55(3):466-70.

19. Miller RT, Nazir N, McDonald T, Cannon CM. The modified rapid emergency medicine score: A novel trauma triage tool to predict in-hospital mortality. Injury. 2017;48(9):1870-7.

20. Kobusingye OC, Lett RR. Hospital-based trauma registries in Uganda. J Trauma. 2000;48(3):498-502.

21. Cassignol A, Marmin J, Cotte J, Cardinale M, Bordes J, Pauly V, et al. Correlation between field triage criteria and the injury severity score of trauma patients in a French inclusive regional trauma system. Scand J Trauma Resusc Emerg Med. 2019;27(1):71.

22. Llompart-Pou JA, Chico-Fernandez M, Sanchez-Casado M, Salaberria-Udabe R, Carbayo-Gorriz C, Guerrero-Lopez F, et al. Scoring severity in trauma: comparison of prehospital scoring systems in trauma ICU patients. Eur J Trauma Emerg Surg. 2017;43(3):351-7.

23. Kondo Y, Abe T, Kohshi K, Tokuda Y, Cook EF, Kukita I. Revised trauma scoring system to predict in-hospital mortality in the emergency department: Glasgow Coma Scale, Age, and Systolic Blood Pressure score. Critical Care. 2011;15(4):R191.

24. Baxt WG, Jones G, Fortlage D. The trauma triage rule: a new, resource-based approach to the prehospital identification of major trauma victims. Ann Emerg Med. 1990;19(12):1401-6.

25. Bond RJ, Kortbeek JB, Preshaw RM. Field trauma triage: combining mechanism of injury with the prehospital index for an improved trauma triage tool. J Trauma. 1997;43(2):283-7.

26. Brown JB, Stassen NA, Bankey PE, Sangosanya AT, Cheng JD, Gestring ML. Mechanism of injury and special consideration criteria still matter: an evaluation of the National Trauma Triage Protocol. J Trauma. 2011;70(1):38-44; discussion -5.

27. Sasser SM, Hunt RC, Sullivent EE, Wald MM, Mitchko J, Jurkovich GJ, et al. Guidelines for field triage of injured patients. Recommendations of the National Expert Panel on Field Triage. MMWR Recomm Rep. 2009;58(Rr-1):1-35.

28. Ciesla DJ, Pracht EE, Tepas JJ, 3rd, Namias N, Moore FA, Cha JY, et al. Measuring trauma system performance: Right patient, right place-Mission accomplished? J Trauma Acute Care Surg. 2015;79(2):263-8.

29. Dinh MM, Bein KJ, Oliver M, Veillard AS, Ivers R. Refining the trauma triage algorithm at an Australian major trauma centre: derivation and internal validation of a triage risk score. Eur J Trauma Emerg Surg. 2014;40(1):67-74.

30. Gray A, Goyder EC, Goodacre SW, Johnson GS. Trauma triage: a comparison of CRAMS and TRTS in a UK population. Injury. 1997;28(2):97-101.

31. Hedges JR, Feero S, Moore B, Haver DW, Shultz B. Comparison of prehospital trauma triage instruments in a semirural population. J Emerg Med. 1987;5(3):197-208.

32. Kane G, Engelhardt R, Celentano J, Koenig W, Yamanaka J, McKinney P, et al. Empirical development and evaluation of prehospital trauma triage instruments. J Trauma. 1985;25(6):482-9.

33. Sacco WJ, Champion HR, Henderson JV, editors. Implementation of severity scores in naval casualty care. 17th Annual Hawaii International Conference on System Sciences; 1984; Hawaii

34. Ornato J, Mlinek EJ, Jr., Craren EJ, Nelson N. Ineffectiveness of the trauma score and the CRAMS scale for accurately triaging patients to trauma centers. Ann Emerg Med. 1985;14(11):1061-4.

35. Knopp R, Yanagi A, Kallsen G, Geide A, Doehring L. Mechanism of injury and anatomic injury as criteria for prehospital trauma triage. Ann Emerg Med. 1988;17(9):895-902.

36. Lerner EB, Shah MN, Swor RA, Cushman JT, Guse CE, Brasel K, et al. Comparison of the 1999 and 2006 trauma triage guidelines: where do patients go? Prehosp Emerg Care. 2011;15(1):12-7.

37. Matsushima K, Chouliaras K, Koenig W, Preston C, Gorospe D, Demetriades D. Should we still use motor vehicle intrusion as a sole triage criterion for the use of trauma center resources? Injury. 2016;47(1):235-8.

38. Morris JA, Jr., Auerbach PS, Marshall GA, Bluth RF, Johnson LG, Trunkey DD. The Trauma Score as a triage tool in the prehospital setting. Jama. 1986;256(10):1319-25.

39. Newgard CD, Fu R, Zive D, Rea T, Malveau S, Daya M, et al. Prospective Validation of the National Field Triage Guidelines for Identifying Seriously Injured Persons. J Am Coll Surg. 2016;222(2):146-58 e2.

40. Sturms LM, Hoogeveen JM, Le Cessie S, Schenck PE, Pahlplatz PV, Hogervorst M, et al. Prehospital triage and survival of major trauma patients in a Dutch regional trauma system: relevance of trauma registry. Langenbecks Arch Surg. 2006;391(4):343-9.

41. Tamim H, Joseph L, Mulder D, Battista RN, Lavoie A, Sampalis JS. Field triage of trauma patients: improving on the Prehospital Index. Am J Emerg Med. 2002;20(3):170-6.

42. Zimmer-Gembeck MJ, Southard PA, Hedges JR, Mullins RJ, Rowland D, Stone JV, et al. Triage in an established trauma system. J Trauma. 1995;39(5):922-8.

43. Gage AM, Traven N, Rivara FP, Jurkovich GJ, Arbabi S. Compliance with Centers for Disease Control and Prevention field triage guidelines in an established trauma system. J Am Coll Surg. 2012;215(1):148-54; discussion 54-6.

44. Newgard CD, Fu R, Lerner EB, Daya M, Jui J, Wittwer L, et al. Role of Guideline Adherence in Improving Field Triage. Prehosp Emerg Care. 2017;21(5):545-55.

45. Sasser SM, Hunt RC, Faul M, Sugerman D, Pearson WS, Dulski T, et al. Guidelines for field triage of injured patients: recommendations of the National Expert Panel on Field Triage, 2011. MMWR Recomm Rep. 2012;61(Rr-1):1-20.

46. Newgard CD, Zive D, Holmes JF, Bulger EM, Staudenmayer K, Liao M, et al. A multisite assessment of the American College of Surgeons Committee on Trauma field triage decision scheme for identifying seriously injured children and adults. J Am Coll Surg. 2011;213(6):709-21.

47. Scheetz LJ. Trends in the accuracy of older person trauma triage from 2004 to 2008. Prehosp Emerg Care. 2011;15(1):83-7.

48. Scheetz LJ. Comparison of type and severity of major injuries among undertriaged and correctly triaged older patients. J Emerg Med. 2012;43(6):1020-8.

49. Newgard CD, Staudenmayer K, Hsia RY, Mann NC, Bulger EM, Holmes JF, et al. The cost of overtriage: more than one-third of low-risk injured patients were taken to major trauma centers. Health Aff (Millwood). 2013;32(9):1591-9.

50. Nishijima DK, Gaona SD, Waechter T, Maloney R, Blitz A, Elms AR, et al. The Incidence of Traumatic Intracranial Hemorrhage in Head-Injured Older Adults Transported by EMS with and without Anticoagulant or Antiplatelet Use. J Neurotrauma. 2018;35(5):750-9.

51. County of Sacramento EMS Agency. County of Sacramento Trauma Triage Criteria 2022 [Available from: <https://dhs.saccounty.gov/PUB/EMS/Documents/PoliciesProceduresProtocols/Effective%20Policies%20July%201%202020/PP-5053%20Trauma%20Triage%20Criteria.pdf>

52. Nishijima DK, Gaona SD, Waechter T, Maloney R, Bair T, Blitz A, et al. Out-of-Hospital Triage of Older Adults With Head Injury: A Retrospective Study of the Effect of Adding "Anticoagulation or Antiplatelet Medication Use" as a Criterion. Ann Emerg Med. 2017;70(2):127-38.e6.

53. Hon S, Gaona SD, Faul M, Holmes JF, Nishijima DK. How Well Do EMS Providers Predict Intracranial Hemorrhage in Head-Injured Older Adults? Prehosp Emerg Care. 2020;24(1):8-14.

54. Parikh PP, Parikh P, Guthrie B, Mamer L, Whitmill M, Erskine T, et al. Impact of triage guidelines on prehospital triage: comparison of guidelines with a statistical model. J Surg Res. 2017;220:255-60.

55. Ichwan B, Darbha S, Shah MN, Thompson L, Evans DC, Boulger CT, et al. Geriatric-specific triage criteria are more sensitive than standard adult criteria in identifying need for trauma center care in injured older adults. Ann Emerg Med. 2015;65(1):92-100 e3.

56. Flottemesch TJ, Raetzman S, Heslin KC, Fingar K, Coffey R, Barrett M, et al. Age-related Disparities in Trauma Center Access for Severe Head Injuries Following the Release of the Updated Field Triage Guidelines. Acad Emerg Med. 2017;24(4):447-57.

57. Barnett AS, Wang NE, Sahni R, Hsia RY, Haukoos JS, Barton ED, et al. Variation in prehospital use and uptake of the national Field Triage Decision Scheme. Prehosp Emerg Care. 2013;17(2):135-48.

58. Davidson GH, Rivara FP, Mack CD, Kaufman R, Jurkovich GJ, Bulger EM. Validation of prehospital trauma triage criteria for motor vehicle collisions. J Trauma Acute Care Surg. 2014;76(3):755-61.

59. Cox S, Currell A, Harriss L, Barger B, Cameron P, Smith K. Evaluation of the Victorian state adult pre-hospital trauma triage criteria. Injury. 2012;43(5):573-81.

60. Kann SH, Hougaard K, Christensen EF. Evaluation of pre-hospital trauma triage criteria: a prospective study at a Danish level I trauma centre. Acta Anaesthesiol Scand. 2007;51(9):1172-7.

61. Lehmann RK, Arthurs ZM, Cuadrado DG, Casey LE, Beekley AC, Martin MJ. Trauma team activation: simplified criteria safely reduces overtriage. Am J Surg. 2007;193(5):630-4; discussion 4-5.

62. Lehmann R, Brounts L, Lesperance K, Eckert M, Casey L, Beekley A, et al. A simplified set of trauma triage criteria to safely reduce overtriage: a prospective study. Arch Surg. 2009;144(9):853-8.

63. Haas B, Gomez D, Zagorski B, Stukel TA, Rubenfeld GD, Nathens AB. Survival of the fittest: the hidden cost of undertriage of major trauma. J Am Coll Surg. 2010;211(6):804-11.

64. Cotte J, Courjon F, Beaume S, Prunet B, Bordes J, N'Guyen C, et al. Vittel criteria for severe trauma triage: Characteristics of over-triage. Anaesth Crit Care Pain Med. 2016;35(2):87-92.

65. Shawhan RR, McVay DP, Casey L, Spears T, Steele SR, Martin MJ. A simplified trauma triage system safely reduces overtriage and improves provider satisfaction: a prospective study. Am J Surg. 2015;209(5):856-62; discussion 62-3.

66. Davis JW, Dirks RC, Sue LP, Kaups KL. Attempting to validate the overtriage/undertriage matrix at a Level I trauma center. J Trauma Acute Care Surg. 2017;83(6):1173-8.

67. Vassallo JM, Smith JE, Wallis LA. Investigating the effects of under-triage by existing major incident triage tools. Eur J Emerg Med. 2019;26(2):139-44.

68. Vassallo J, Beavis J, Smith JE, Wallis LA. Major incident triage: Derivation and comparative analysis of the Modified Physiological Triage Tool (MPTT). Injury. 2017;48(5):992-9.

69. Báez AA, Lane PL, Sorondo B. System compliance with out-of-hospital trauma triage criteria. J Trauma. 2003;54(2):344-51.

70. Candefjord S, Buendia R, Caragounis EC, Sjoqvist BA, Fagerlind H. Prehospital transportation decisions for patients sustaining major trauma in road traffic crashes in Sweden. Traffic Inj Prev. 2016;17 Suppl 1:16-20.

71. Widgren BR, Jourak M. Medical Emergency Triage and Treatment System (METTS): a new protocol in primary triage and secondary priority decision in emergency medicine. J Emerg Med. 2011;40(6):623-8.

72. Ciesla DJ, Pracht EE, Leitz PT, Spain DA, Staudenmayer KL, Tepas JJ, 3rd. The trauma ecosystem: The impact and economics of new trauma centers on a mature statewide trauma system. J Trauma Acute Care Surg. 2017;82(6):1014-22.

73. Di Bartolomeo S, Sanson G, Michelutto V, Nardi G, Burba I, Francescutti C, et al. Epidemiology of major injury in the population of Friuli Venezia Giulia-Italy. Injury. 2004;35(4):391-400.

74. Faul M, Xu L, Sasser SM. Hospitalized Traumatic Brain Injury: Low Trauma Center Utilization and High Interfacility Transfers among Older Adults. Prehosp Emerg Care. 2016;20(5):594-600.

75. Garwe T, Stewart K, Stoner J, Newgard CD, Scott M, Zhang Y, et al. Out-of-hospital and Inter-hospital Under-triage to Designated Tertiary Trauma Centers among Injured Older Adults: A 10-year Statewide Geospatial-Adjusted Analysis. Prehosp Emerg Care. 2017;21(6):734-43.

76. Haas B, Stukel TA, Gomez D, Zagorski B, De Mestral C, Sharma SV, et al. The mortality benefit of direct trauma center transport in a regional trauma system: a population-based analysis. J Trauma Acute Care Surg. 2012;72(6):1510-5; discussion 5-7.

77. Hsia RY, Wang E, Torres H, Saynina O, Wise PH. Disparities in trauma center access despite increasing utilization: data from California, 1999 to 2006. J Trauma. 2010;68(1):217-24.

78. Kodadek LM, Selvarajah S, Velopulos CG, Haut ER, Haider AH. Undertriage of older trauma patients: is this a national phenomenon? J Surg Res. 2015;199(1):220-9.

79. American College of Surgeons Committee on Trauma. Resources for the Optimal Care of the Injured Patient (2014 Standards). Chicago: American College of Surgeons; 2014.

80. Kreis DJ, Jr., Fine EG, Gomez GA, Eckes J, Whitwell E, Byers PM. A prospective evaluation of field categorization of trauma patients. J Trauma. 1988;28(7):995-1000.

81. Champion HR. Field triage of trauma patients. Ann Emerg Med. 1982;11(3):160-1.

82. Lale A, Krajewski A, Friedman LS. Undertriage of Firearm-Related Injuries in a Major Metropolitan Area. JAMA surgery. 2017;152(5):467-74.

83. Osler T, Baker SP, Long W. A modification of the injury severity score that both improves accuracy and simplifies scoring. J Trauma. 1997;43(6):922-5; discussion 5-6.

84. Long WB, Bachulis BL, Hynes GD. Accuracy and relationship of mechanisms of injury, trauma score, and injury severity score in identifying major trauma. Am J Surg. 1986;151(5):581-4.

85. Newgard CD, Hedges JR, Diggs B, Mullins RJ. Establishing the need for trauma center care: anatomic injury or resource use? Prehosp Emerg Care. 2008;12(4):451-8.

86. Newgard CD, Fu R, Lerner EB, Daya M, Wright D, Jui J, et al. Deaths and high-risk trauma patients missed by standard trauma data sources. J Trauma Acute Care Surg. 2017;83(3):427-37.

87. Rubenson Wahlin R, Ponzer S, Skrifvars MB, Lossius HM, Castren M. Effect of an organizational change in a prehospital trauma care protocol and trauma transport directive in a large urban city: a before and after study. Scand J Trauma Resusc Emerg Med. 2016;24:26.

88. Santaniello JM, Esposito TJ, Luchette FA, Atkian DK, Davis KA, Gamelli RL. Mechanism of injury does not predict acuity or level of service need: field triage criteria revisited. Surgery. 2003;134(4):698-703; discussion -4.

89. Scheetz LJ. Trauma center versus non-trauma center admissions in adult trauma victims by age and gender. Prehosp Emerg Care. 2004;8(3):268-72.

90. Tamim H, Joseph L, Mulder D, Battista RN, Lavoie A, Sampalis JS. Field triage of trauma patients: Improving on the Prehospital Index. The American journal of emergency medicine. 2002;20(3):170-6.

91. Twijnstra MJ, Moons KGM, Simmermacher RKJ, Leenen LPH. Regional Trauma System Reduces Mortality and Changes Admission Rates: A Before and After Study. Annals of surgery. 2010;251(2):339-43.

92. West JG, Murdock MA, Baldwin LC, Whalen E. A method for evaluating field triage criteria. J Trauma. 1986;26(7):655-9.

93. Andrews R, Wynn MT, Vallmuur K, Elcock M, Rashford S, Bosley E, et al. Trauma by-pass guideline: A data-driven conformance analysis for road trauma cases in Queensland. Emergency medicine Australasia : EMA. 2021;33(6):1059-65.

94. Clinical Quality & Patient Safety Unit Queensland Ambulance Service. Clinical Practice Guideline: Trauma/Pre-hospital Trauma By-pass. Brisbane: Queensland Ambulance Service; 2019.

95. Atiksawedparit P, Rattanasiri S, Sittichanbuncha Y, McEvoy M, Suriyawongpaisal P, Attia J, et al. Prehospital prediction of severe injury in road traffic injuries: A multicenter cross-sectional study. Injury. 2019;50(9):1499-506.

96. Bischoff T, Briton J, Baumber B, Lewell M, Nolan B. A review of secondary interfacility trauma transfers meeting provincial prehospital trauma triage guidelines. CJEM. 2021;23(6):837-41.

97. Ontario Ministry of Health. Emergency Health Regulatory and Accountability Branch Ministry of Health. Basic Life Support Patient Care Standard. Vol.3.3. Ontario: Queen's Printer for Ontario; 2021.

98. Bosson N, Kaji AH, Gausche-Hill M, Kim D, Putnam B, Schlesinger S, et al. Evaluation of Trauma Triage Criteria Performance in a Regional Trauma System. Prehospital emergency care : official journal of the National Association of EMS Physicians and the National Association of State EMS Directors. 2019;23(6):828-37.

99. Los Angeles County EMS Agency. Reference No. 506.1 Trauma Triage Decision Scheme. Los Angeles: Los Angeles County EMS Agency,. 2012.

100. Fuller G, Keating S, Turner J, Miller J, Holt C, Smith JE, et al. Injured patients who would benefit from expedited major trauma centre care: a consensus-based definition for the United Kingdom. British paramedic journal. 2021;6(3):7-14.

101. Hansen J, Rasmussen LS, Steinmetz J. Prehospital triage of trauma patients before and after implementation of a regional triage guideline. Injury. 2022;53(1):54-60.

102. Yeung WH, Wong John KS, Tsui KL, Lam Tommy SK, Lui CT, Lau CL. Can mechanism of injury improve trauma diversion? A retrospective cross-sectional study. Hong Kong Journal of Emergency Medicine. 2022.

103. Leung GKK. Trauma system in Hong Kong. Surgical practice. 2010;14(2):38-43.

104. Bagnato C, Ranzato K, Giarraca A, Restelli P, Saronni S, Gadda G, et al. A prospective study comparing two methods of pre-hospital triage for trauma. Updates in surgery. 2022;74(5):1739-47.

105. Benhamed A, Fraticelli L, Claustre C, Gossiome A, Cesareo E, Heidet M, et al. Risk factors and mortality associated with undertriage after major trauma in a physician-led prehospital system: a retrospective multicentre cohort study. European journal of trauma and emergency surgery : official publication of the European Trauma Society. 2022;49(4):1707-15.

106. Cassignol A, Markarian T, Cotte J, Marmin J, Nguyen C, Cardinale M, et al. Evaluation and Comparison of Different Prehospital Triage Scores of Trauma Patients on In-Hospital Mortality. Prehospital emergency care : official journal of the National Association of EMS Physicians and the National Association of State EMS Directors. 2019;23(4):543-50.

107. Jeong JH, Park YJ, Kim DH, Kim TY, Kang C, Lee SH, et al. The new trauma score (NTS): a modification of the revised trauma score for better trauma mortality prediction. BMC Surg. 2017;17(1):77.

108. Deeb A-P, Phelos HM, Peitzman AB, Billiar TR, Sperry JL, Brown JB. Making the call in the field: Validating emergency medical services identification of anatomic trauma triage criteria. The journal of trauma and acute care surgery. 2021;90(6):967-72.

109. Escobar N, DiMaggio C, Frangos SG, Winchell RJ, Bukur M, Klein MJ, et al. Disparity in Transport of Critically Injured Patients to Trauma Centers: Analysis of the National Emergency Medical Services Information System (NEMSIS). Journal of the American College of Surgeons. 2022;235(1):78-85.

110. Fernandez AR, Bourn SS, Hall GD, Crowe RP, Myers JB. Patient Outcomes Based on the 2011 CDC Guidelines for Field Triage of Injured Patients. Journal of trauma nursing : the official journal of the Society of Trauma Nurses. 2023;30(1):5-13.

111. Kang BH, Jung K, Kim S, Youn SH, Song SY, Huh Y, et al. Accuracy and influencing factors of the Field Triage Decision Scheme for adult trauma patients at a level-1 trauma center in Korea. BMC emergency medicine. 2022;22(1):101.

112. Leichtle SW, Poulos NG, Whelan J, Aboutanos M. Field triage of motor vehicle crashes: Which factors predict high injury severity? American Surgeon. 2019;85(7):764-7.

113. Mohammed Z, Saleh Y, AbdelSalam EM, Mohammed NBB, El-Bana E, Hirshon JM. Evaluation of the Revised Trauma Score, MGAP, and GAP scoring systems in predicting mortality of adult trauma patients in a low-resource setting. BMC emergency medicine. 2022;22(1):90.

114. Norouzi V, Feizi I, Vatankhah S, Pourshaikhian M. Calculation of the probability of survival for trauma patients based on trauma score and the injury severity score model in fatemi hospital in ardabil. Arch Trauma Res. 2013;2(1):30-5.

115. Moran ME, Nash JE. Revised Trauma Scale StatPearls: StatPearls Publishing; 2021.

116. Newgard CD, Fischer PE, Gestring M, Michaels HN, Jurkovich GJ, Lerner EB, et al. National guideline for the field triage of injured patients: Recommendations of the National Expert Panel on Field Triage, 2021. The journal of trauma and acute care surgery. 2022;93(2):e49-e60.

117. Newgard CD, Lin A, Eckstrom E, Caughey A, Malveau S, Griffiths D, et al. Comorbidities, anticoagulants, and geriatric-specific physiology for the field triage of injured older adults. J Trauma Acute Care Surg. 2019;86(5):829-37.

118. Shanahan TAG, Fuller GW, Sheldon T, Turton E, Quilty FMA, Marincowitz C. External validation of the Dutch prediction model for prehospital triage of trauma patients in South West region of England, United Kingdom. Injury. 2021;52(5):1108-16.

119. van Rein EAJ, van der Sluijs R, Voskens FJ, Lansink KWW, Houwert RM, Lichtveld RA, et al. Development and Validation of a Prediction Model for Prehospital Triage of Trauma Patients. JAMA surgery. 2019;154(5):421-9.
