# Supplementary material for: Substantial heterogeneity in trauma triage tool characteristic operationalization for identification of major trauma: a hybrid systematic review
Source: Eur J Trauma Emerg Surg. 2025 Jan 24;51(1):74. doi: 10.1007/s00068-024-02694-6 (PMC11842439; doi:10.1007/s00068-024-02694-6)
Supplement: Supplementary file 5 — Supplementary Material 5 [file 68_2024_2694_MOESM5_ESM.docx]

**Appendix 5: Registration and protocol**

This hybrid review was registered with the International Prospective Register of Systematic Reviews (PROSPERO) database (Protocol Number: CRD42023393094). The protocol for the review was published in HRB Open (1). There were a number of deviations to information provided in the protocol. Resource and time constraints meant it was not possible to assess the quality of studies included in the review. As the objective of the review was examine the prehospital characteristics applied in the international literature, we needed as extensive a range of tools included as was possible. The quality of the studies would not have impacted the study findings given it was important that as many prehospital characteristics from triage tools were included, irrespective of the quality of the tools.

Secondly, the protocol specified that studies confined to a specific population (paediatrics or older patient populations) would be included in the review and analysed by separating out those tools that are specific to populations. However, it was subsequently decided to exclude studies which were confined to paediatric and older patient populations. There were a number of reasons for this. Firstly, as the review was conducted to inform the development of a clinical prediction tool for the general trauma patient population, it was important that the evidence informing the development of the clinical prediction tool was appropriate to the patient population to which it would be applied. In tandem with this, paediatric and older patient populations have differing physiological characteristics, for example the general systolic blood pressure and respiratory rate for young children and adults over the age of 65 are considerably different (2-6). These groups also often present with specific and complex care needs. Thus, there is a considerable and growing body of literature to suggest trauma triaging in paediatric or older adult populations should include criteria that is more sensitive to standard adult criteria in identifying major trauma patients that would benefit from care in a major trauma centre (2-6).

Finally, we had hoped to examine alternative definitions of major trauma in the analysis. However, following data extraction it became apparent that this would not be possible given that the majority of studies applied the Injury Severity Score (ISS) to define major trauma patients. The Trauma and Injury Severity Score (TRISS) (7) was only applied in one study and the New Injury Severity Score (NISS) (8) was only applied in two studies.

**References**

1. Donnelly N, Linvill M, Zaidan R, Simpson A, Brent L, Hickey P, et al. Prehospital characteristics that identify major trauma patients: A hybrid systematic review protocol [version 2; peer review: 1 approved, 1 approved with reservations]. HRB Open Research. 2023;6(31).

2. Brown JB, Gestring ML, Forsythe RM, Stassen NA, Billiar TR, Peitzman AB, et al. Systolic blood pressure criteria in the National Trauma Triage Protocol for geriatric trauma: 110 is the new 90. J Trauma Acute Care Surg. 2015;78(2):352-9.

3. Newgard CD, Lin A, Eckstrom E, Caughey A, Malveau S, Griffiths D, et al. Comorbidities, anticoagulants, and geriatric-specific physiology for the field triage of injured older adults. J Trauma Acute Care Surg. 2019;86(5):829-37.

4. Ichwan B, Darbha S, Shah MN, Thompson L, Evans DC, Boulger CT, et al. Geriatric-specific triage criteria are more sensitive than standard adult criteria in identifying need for trauma center care in injured older adults. Ann Emerg Med. 2015;65(1):92-100 e3.

5. Ardolino A, Cheung CR, Lawrence T, Bouamra O, Lecky F, Berry K, et al. The accuracy of existing prehospital triage tools for injured children in England: an analysis using emergency department data. Emerg Med J. 2015;32(5):397-400.

6. Parikh PP, Parikh P, Guthrie B, Mamer L, Whitmill M, Erskine T, et al. Impact of triage guidelines on prehospital triage: comparison of guidelines with a statistical model. J Surg Res. 2017;220:255-60.

7. Llompart-Pou JA, Chico-Fernandez M, Sanchez-Casado M, Salaberria-Udabe R, Carbayo-Gorriz C, Guerrero-Lopez F, et al. Scoring severity in trauma: comparison of prehospital scoring systems in trauma ICU patients. Eur J Trauma Emerg Surg. 2017;43(3):351-7.

8. Osler T, Baker SP, Long W. A modification of the injury severity score that both improves accuracy and simplifies scoring. J Trauma. 1997;43(6):922-5; discussion 5-6.
